# A Dual Regulatory Role of the PhoU Protein in Salmonella Typhimurium

**DOI:** 10.1128/mbio.00811-22

**Published:** 2022-05-31

**Authors:** Soomin Choi, Gyunghwa Jeong, Eunna Choi, Eun-Jin Lee

**Affiliations:** a Department of Life Sciences, School of Life Sciences and Biotechnology, Korea University, Seoul, South Korea; National Institute of Child Health and Human Development (NICHD)

**Keywords:** phosphate limitation, low Mg^2+^, *pst* phosphate-transport system, PhoB/PhoR two-component system, phosphate limitation

## Abstract

Bacteria utilize two-component regulatory systems to sense and respond to their surroundings. Unlike other two-component systems that directly sense through a sensory domain in the histidine kinase (HK), the PhoB/PhoR two-component system requires additional proteins, including the PstSCAB phosphate transporter and the PhoU protein, to sense phosphate levels. Although PhoU is involved in phosphate signaling by connecting the PstSCAB transporter and PhoR histidine kinase, the mechanism by which PhoU controls expression of *pho* regulon genes has not yet been clearly understood. Here, we identified PhoU residues required for interacting with PhoR histidine kinase from the intracellular pathogen Salmonella enterica serovar Typhimurium. The PhoU Ala147 residue interacts with the PhoR PAS domain and is involved in repressing *pho* expression in high phosphate. Unexpectedly, the PhoU Arg184 residue interacts with the PhoR histidine kinase domain and is required for activating *pho* expression in low Mg^2+^ by increasing PhoR autophosphorylation, revealing its new function. The substitution of the Arg184 to Gly codon decreased Salmonella virulence both in macrophages and in mice, suggesting that PhoU’s role in promoting PhoR autophosphorylation is required during Salmonella infection.

## INTRODUCTION

Inorganic phosphate (P_i_) is one of the essential elements involved in many biological processes (1). It needs to be incorporated as a component of macromolecules, including nucleic acids, membranes, and phosphate-containing organic molecules. Additionally, it stores energy in the form of a high-energy phosphate group which can be transferred during many enzymatic reactions and transduces information via transferring the phosphate group in signaling pathways. Therefore, living organisms constantly sense and respond to phosphate levels. In Gram-negative bacteria, phosphate levels are sensed and controlled by the PhoB/PhoR two-component system, which has been well studied in Escherichia coli (2). PhoR is a membrane-bound histidine kinase (HK) that consists of a transmembrane (TM) domain with two helices, a Per-Arnt-Sim (PAS) domain, a dimerization and histidine phosphotransfer (DHp) domain, and a catalytic active/ATP-binding (CA) domain (3). In phosphate-limiting conditions, PhoR self-phosphorylates the histidine residue in its own DHp domain and transfers the phosphate group to the aspartate residue of the cognate PhoB response regulator. Phosphorylated PhoB then binds to the promoters of PhoB-dependent genes and increases transcription of the so-called *pho* regulon genes, including the PstSCAB_2_ high-affinity phosphate transporter, the PhoU accessory protein, and the PhoB/PhoR two-component system itself (3). Phosphate signaling via PhoR histidine kinase is unique because, unlike other histidine kinases that contain a periplasmic sensory domain involved in sensing a corresponding signal(s), PhoR lacks such a periplasmic domain. Rather, it was suggested that the phosphate-transporting activity through the PstSCAB transporter is linked to the kinase activity of PhoR histidine kinase (4). In Escherichia coli, the connection between the PhoB/PhoR two-component system and the PstSCAB_2_ phosphate transporter is physically linked by the PhoU protein that is loosely associated with the inner membrane (5, 6). Even though the PhoU protein bridges PhoR histidine kinase and the PstSCAB_2_ phosphate transporter, it was reported that the interaction with the former is stronger than that with the latter (5). A previous bacterial two-hybrid analysis determined that, more specifically, PhoU binds to the PAS domain of the PhoR kinase (5). Given that the PAS domain generally functions as a molecular sensor by interacting with other molecules or proteins (7, 8), molecular interactions between PstB, PhoU, and the PhoR PAS domain appear to be critical in phosphate signaling.

The function of the PhoU protein has been suggested as a negative regulator in phosphate signaling based on the fact that, in E. coli and other bacteria, deletion of the *phoU* gene constitutively derepressed expression of *pho* regulon genes in both high- and low-phosphate conditions (9–12). The elevated expression of *pho* regulon genes in the *phoU* deletion mutant suggests that the autophosphorylation activity of the PhoR histidine kinase is always active unless PhoU binds to PhoR and represses autophosphorylation activity. Although it is not formally demonstrated, this also led to an assumption that PhoU might be dissociated/released from the PhoR histidine kinase in low-phosphate conditions to promote autophosphorylation activity of PhoR, resulting in activation of *pho* expression. However, it is still unclear how PhoU accommodates its conformation within the PstSCAB-PhoU-PhoR signaling complex in phosphate-limiting or -replete conditions.

Interestingly, a previous direct coupling analysis suggested that the E. coli PhoU protein appears to be proximal to the PhoR CA domain in addition to the above-mentioned PAS domain (6). This implies that PhoU could be in close contact with PhoR histidine kinase at multiple sites, although the physiological implications of such interactions are unclear. In addition, several recent studies have reported that PhoU might have an additional role(s) other than a negative regulator in phosphate signaling. When E. coli is grown in low-phosphate and low-potassium media, PhoU is likely to connect the phosphate-sensing PhoB/PhoR two-component system and the potassium-sensing KdpE/KdpD two-component system (13). Thus, in the absence of the cognate KdpD histidine kinase, PhoU allows expression of KdpE-dependent potassium transporter genes in response to a phosphorelay via PhoR histidine kinase (13). PhoU also promotes mutagenesis in E. coli when a double-strand break becomes mutagenic in a strain activating SOS response and stationary sigma factor (σ^S^)-mediated response (14). However, this mutagenic function of PhoU seems to be linked not to phosphate levels but the ArcA histidine kinase-dependent response controlling aerobic respiration (14). Moreover, PhoU from Caulobacter crescentus was reported that it is not involved in repressing *pho* signaling in high-phosphate conditions, but it is involved in polyphosphate accumulation (10). Cumulatively, it implies that PhoU might have multiple functions that need to be addressed.

**FIG 1:**
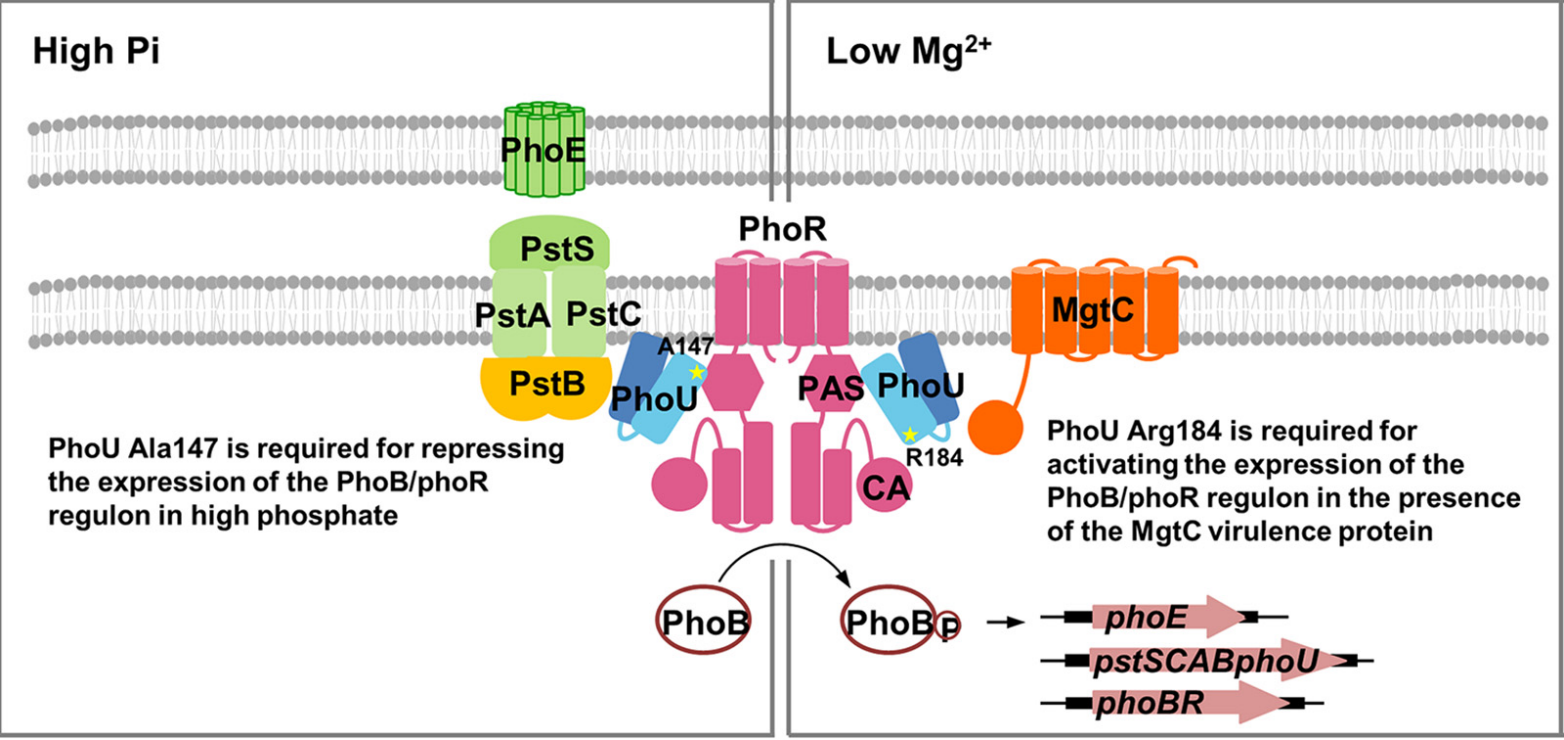
The dual regulatory role of the PhoU protein in Salmonella enterica. In high phosphate, PhoU bridges between the PhoR histidine kinase and the PstB phosphate import ATP-binding protein and represses the PhoR histidine kinase to repress expression of PhoB-dependent genes that include the *phoE* phosphoporin gene. The PhoU Ala147 residue is required for interacting with the PhoR PAS domain, and the removal of the interaction by substituting Ala147 to Glu derepresses *phoE* expression, even in high-phosphate conditions. In low Mg^2+^, Salmonella produces the MgtC virulence protein that binds to the PhoR CA domain and activates expression of PhoB-dependent genes independent of the phosphate availability (16). The PhoU Arg184 residue is required for interacting with the PhoR CA domain, and the removal of the interaction by substituting Arg184 to Gly prevents MgtC-mediated PhoR autophosphorylation and subsequent induction of *phoE* mRNA levels. PstB is a part of *pst* high-affinity phosphate-specific transport system together with PstS phosphate-binding protein and two membrane permeases, PstA and PstC proteins.

In the intracellular pathogen Salmonella enterica serovar Typhimurium, the *pst* and *phoB*/*phoR* genes were highly induced when Salmonella was inside macrophages (15). In addition, Salmonella virulence protein MgtC binds to the CA domain of PhoR and activates PhoR autophosphorylation, thereby promoting expression of *pho* regulon genes and phosphate uptake (16). Because the *mgtC* gene is also highly expressed inside macrophages (15, 17), high levels of *mgtC* expression support the activation of the PhoB/PhoR two-component system during Salmonella infection, the underlying mechanism of which remains unclear (16, 18). Here, we investigated the roles of the PhoU protein from Salmonella enterica serovar Typhimurium. Although maintaining phosphate levels appears to be critical for Salmonella pathogenesis (16, 19), the role(s) of the PhoU protein in *pho* expression and Salmonella pathogenesis has not been investigated yet. Based on protein homology modeling, we identified the PhoU residues that are required for PhoR interaction (Fig. 1). Salmonella PhoU Ala147 residue was identified to interact with the PAS domain of PhoR, similar to that previously reported in E. coli. Interestingly, we found another residue, PhoU Arg184, which is required for the HK domain of PhoR histidine kinase. Substitutions of each residue in PhoU showed a different impact on expression of *pho* regulon genes depending on growth conditions, revealing an unexpected role of PhoU in controlling expression of *pho* regulon genes.

## RESULTS

### The Ala147 residue of Salmonella PhoU is required to interact with PhoR histidine kinase and represses expression of the PhoB-dependent genes in high phosphate.

**FIG 2:**
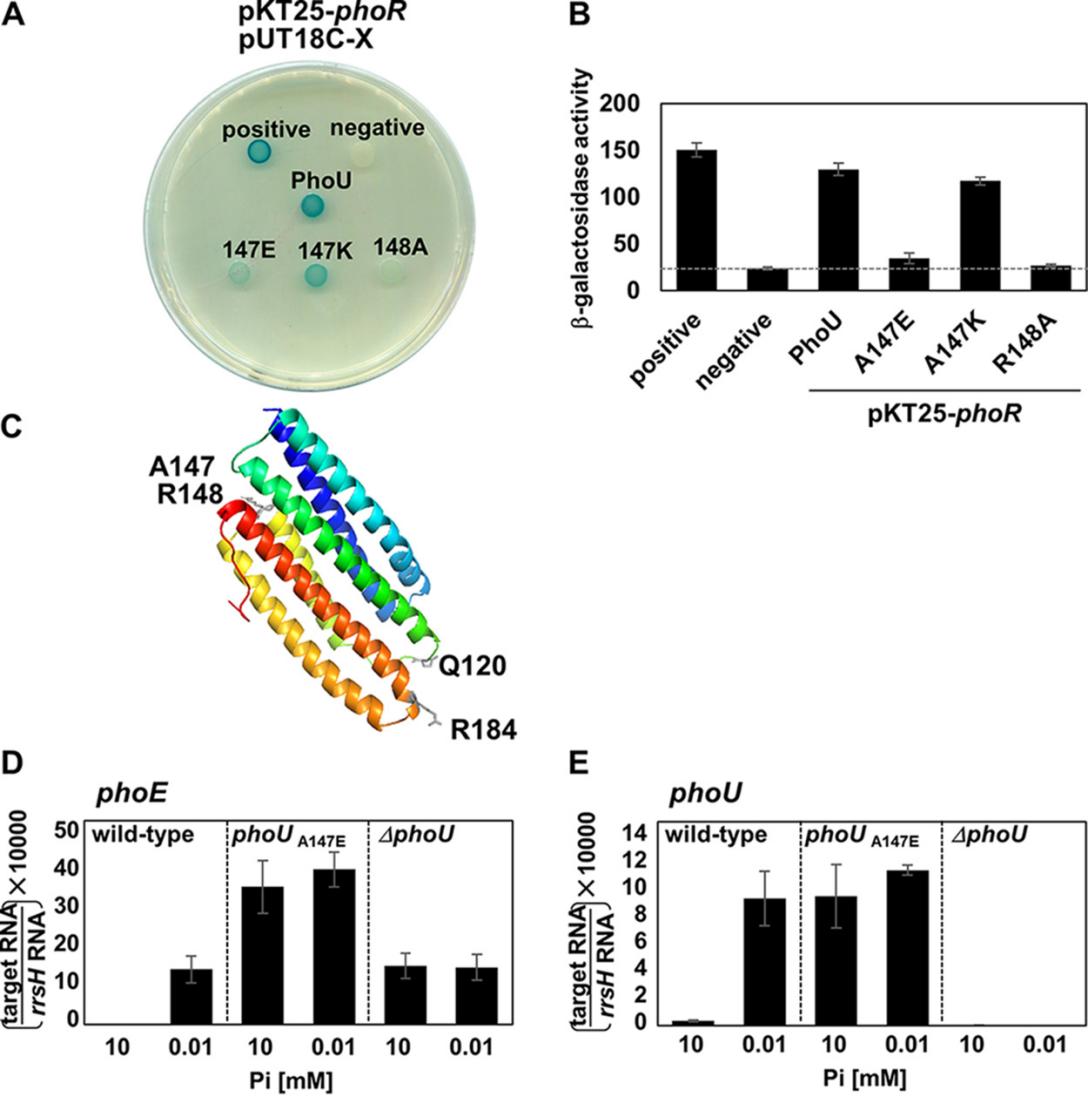
PhoU Ala147 and Arg148 residues are required for PhoR interaction and suppression of *phoE* mRNA levels in high phosphate. (A) Bacterial two-hybrid assay between PhoR and PhoU or PhoU variant proteins. Escherichia coli BTH101 strains harboring two plasmids, pUT18c and pKT25 derivatives, expressing an N-terminal fusion of the *cyaA* T25 fragment to the *phoR* gene and N-terminal fusions of the *cyaA* T18 fragment to the wild-type *phoU*, *phoU*^A147E^, *phoU*^A147K^, and *phoU*^R148A^ genes or the pUT18c empty vector (negative) were spotted as indicated. Cells expressing both pUT18-*mgtC* and pKT25-*mgtR* are spotted as a positive control (37). Cells were spotted onto LB plates containing 80 μM X-Gal and 0.1 mM IPTG and incubated at 30°C for 40 h. Blue-colored colonies indicate a positive interaction. (B) β-Galactosidase assay from strains listed above. The average β-galactosidase activities (Miller units) are shown as mean ± SD (*n* = 3, independent measurements). (C) Modeled structure of the Salmonella PhoU monomer predicted from a homology modeling program (Phyre2) based on the crystal structure of Pseudomonas aeruginosa PhoU (PDB ID 4Q25). Arg184 is located at the α6 helix and Gln120 is located at the α3-α4 loop. The Arg184 and Gln120 residues are indicated with gray sticks, and PAS domain-interacting residues (Ala147 and Arg148) are located between α4 and α5 regions and indicated with pale gray sticks (6). The protein is colored blue to red, going from the amino terminus to the carboxyl terminus. (D and E) Relative mRNA levels of the *phoE* (D) and *phoU* (E) genes in Salmonella strains with the wild-type *phoU* gene (14028s), Ala147 to Glu-substituted *phoU* gene (SM323), or a strain that deleted the *phoU* gene. Bacteria were grown for 5 h in N-minimal medium containing 10 mM P_i_ (high P_i_) or 0.01 mM P_i_ (low P_i_). Shown are the means ± SD (*n* = 3, independent measurements). Relative mRNA levels represent (target RNA/*rrsH* RNA) × 10,000.

In E. coli, a previous study reported that PhoU interacts with the PAS domain of PhoR histidine kinase to repress expression of PhoB-dependent genes in high-phosphate conditions (5, 20). To explore a functional role of PhoU in Salmonella enterica, we started to test whether Salmonella PhoU could interact with PhoR histidine kinase using a bacterial two-hybrid assay. Indeed, the E. coli *cyaA* mutant expressing T18-PhoU and T25-PhoR together exhibited a strong blue color on Luria-Bertani (LB) X-Gal (5-bromo-4-chloro-3-indolyl-β-d-galactopyranoside) plates, indicating that N-terminally T18-fused PhoU and N-terminally T25-fused PhoR proteins physically interact with each other to complement CyaA adenylate cyclase and produce β-galactosidase from a cAMP-dependent promoter (Fig. 2A and B).

We then tested whether Ala147 and Arg148 residues of the PhoU protein are required for PhoR interaction based on the following reasons. First, the substitutions of the E. coli PhoU Ala147 or Arg148 residues were reported to weaken the interaction with PhoR, losing the ability to suppress the elevated alkaline phosphatase activity of the chromosomal *phoU* deletion mutant when expressed heterologously (6). Second, homology-based modeling and amino acid sequence alignment suggested that Ala147 and Arg148 residues in the PhoU proteins from E. coli and S. enterica are well conserved in both species (Fig. 2C; see Fig. S1 in the supplemental material). Similar to those reported in E. coli, T18-PhoU derivatives that harbor the Ala147-to-Glu or Arg148-to-Ala substitutions significantly decreased β-galactosidase production when coexpressed with T25-PhoR (Fig. 2A and B), indicating that Ala147 and Arg148 residues in the Salmonella PhoU protein are required for PhoR interaction. It is interesting to note that the Ala147-to-Lys substitution did not affect the interaction (Fig. 2A and B), unlike the E. coli counterpart (6). When we created a *phoU* mutant strain with the Ala147-to-Glu substitution at its chromosomal location, the *phoU* variant with the Ala147-to-Glu substitution completely lost the ability to repress mRNA levels of the *phoE* and *phoU* genes in high phosphate (Fig. 2D and E), supporting the notion that Salmonella PhoU is a negative regulator in phosphate signaling and PhoU Ala147 and Arg148 residues are required for this activity by interacting with PhoR, possibly via the PAS domain.

10.1128/mbio.00811-22.1 FIG S1 Ala147, Arg148, and Arg184 residues in Salmonella PhoU are conserved in other bacteria. (A to D) Homology-modeled structures of the PhoU proteins from Salmonella enterica serovar Typhimurium 14028s (SALT1) (A), Escherichia coli K-12 substr. MG1655 (ECOLI) (B) Yersinia enterocolitica subsp. enterocolitica 8081 (YERE8) (C), and PhoY1 protein from Mycobacterium tuberculosis H37Rv (MYCTU) (D). Ala147 (Val140 for Mycobacterium tuberculosis PhoY1) and Arg184 (Arg174 for M. tuberculosis PhoY1) residues are indicated. All proteins are colored blue to red, going from the amino terminus to the carboxyl terminus. (E) Alignment of the amino acid sequences of the PhoU proteins from strains listed above. Ala147 (Val140 for M. tuberculosis PhoY1) and Arg148 (Asn141 for M. tuberculosis PhoY1) residues are colored in pink, and Arg184 (Arg174 for M. tuberculosis PhoY1) residues are colored in blue. Download FIG S1, TIF file, 1.8 MB. Copyright © 2022 Choi et al. 2022 Choi et al. https://creativecommons.org/licenses/by/4.0/ This content is distributed under the terms of the Creative Commons Attribution 4.0 International license.

Given that the PhoU-PhoR interaction was recapitulated with the PhoR PAS domain alone in E. coli (5), one can expect that this is also true for Salmonella. However, the PAS domain of PhoR alone was not sufficient for interacting with PhoU when several T25-PhoR subclones were tested to identify the region(s) required for PhoU interaction (Fig. S2). Only the HK domain exhibited a weak interaction when we incubated for 60 h (Fig. S2D). This suggests that the PhoU-PhoR interaction may require additional residues or regions.

10.1128/mbio.00811-22.2 FIG S2 None of the domains of the PhoR protein are sufficient for PhoU interaction. (A) Schematic representation of the domain structure of the PhoR protein, including a transmembrane (TM) domain, a Per-Arnt-Sim (PAS) domain, a dimerization histidine phosphotransfer (DHp) domain, and a catalytic/ATP-binding (AP) domain. The HK domain includes DHp and CA domains. (B) Bacterial two-hybrid assay between the PhoU and full-length PhoR protein or its domains. Escherichia coli BTH101 strains harboring two plasmids (pUT18 and pKT25 derivatives) expressing the C-terminal fusion of the *cyaA* T18 fragment to the *phoU* coding region and N-terminal fusions of the *cyaA* T25 fragment to either the coding regions of the full-length *phoR* (PhoR), *phoR*_1–60_ (TM), *phoR*_61–180_ (PAS), *phoR*_181–431_ (DHp+CA), *phoR*_1–180_ (TM+PAS), and *mgtR* (positive) genes of the pKT25 empty vector (negative) are indicated. Cells were spotted onto LB plates containing 80 μM X-Gal and 0.1 mM IPTG and incubated at 30°C for 40 h. Blue-colored colonies indicate a positive interaction. (C) β-Galactosidase assay from strains listed above. The average β-galactosidase activities (Miller units) are shown as mean ± SD (*n* = 3, independent measurements). (D) The PhoR HK domain alone exhibits a weak interaction with PhoU. Bacterial two-hybrid assay between the full-length or HK domain of PhoR and PhoU variant proteins. Escherichia coli BTH101 strains harboring two plasmids, pUT18c and pKT25 derivatives, expressing N-terminal fusions of the *cyaA* T18 fragment to the wild-type *phoU, phoU*^R184A^, or *phoU*^R184G^ genes and N-terminal fusions of the *cyaA* T25 fragment to either the full-length (PhoR) or the histidine kinase domain (PhoR^HK^) of *phoR* genes and the pKT25 empty vector (negative) were spotted onto MacConkey plates containing 1% maltose and 0.5 mM IPTG and incubated at 30°C for 60 h. Cells expressing both pUT18-*mgtC* and pKT25-*mgtR* are spotted as a positive control. Blue-colored colonies indicate a positive interaction. (E) β-Galactosidase assay from strains listed above. The average β-galactosidase activities (Miller units) are shown as mean ± SD (*n* = 3, independent measurements). (F and G) The C-terminally GFP-tagged PhoR HK domain immunoprecipitates PhoU-FLAG. (F) Crude extracts prepared from Salmonella strains with the C-terminally FLAG-tagged *phoU* gene, *phoU*^R184A^, or *phoU*^R184G^ genes expressing either PhoR^HK^-GFP or the empty vector (pTGFP) were detected with anti-GFP (top) and anti-FLAG (bottom) antibodies. (G) Eluted fractions prepared from strains listed above were detected with anti-GFP (top) and anti-FLAG (bottom) antibodies after immunoprecipitation with anti-GFP antibody-coated beads. Download FIG S2, TIF file, 1.6 MB. Copyright © 2022 Choi et al. 2022 Choi et al. https://creativecommons.org/licenses/by/4.0/ This content is distributed under the terms of the Creative Commons Attribution 4.0 International license.

### A region that includes the α-helix 6 of PhoU is required to interact with PhoR histidine kinase.

**FIG 3:**
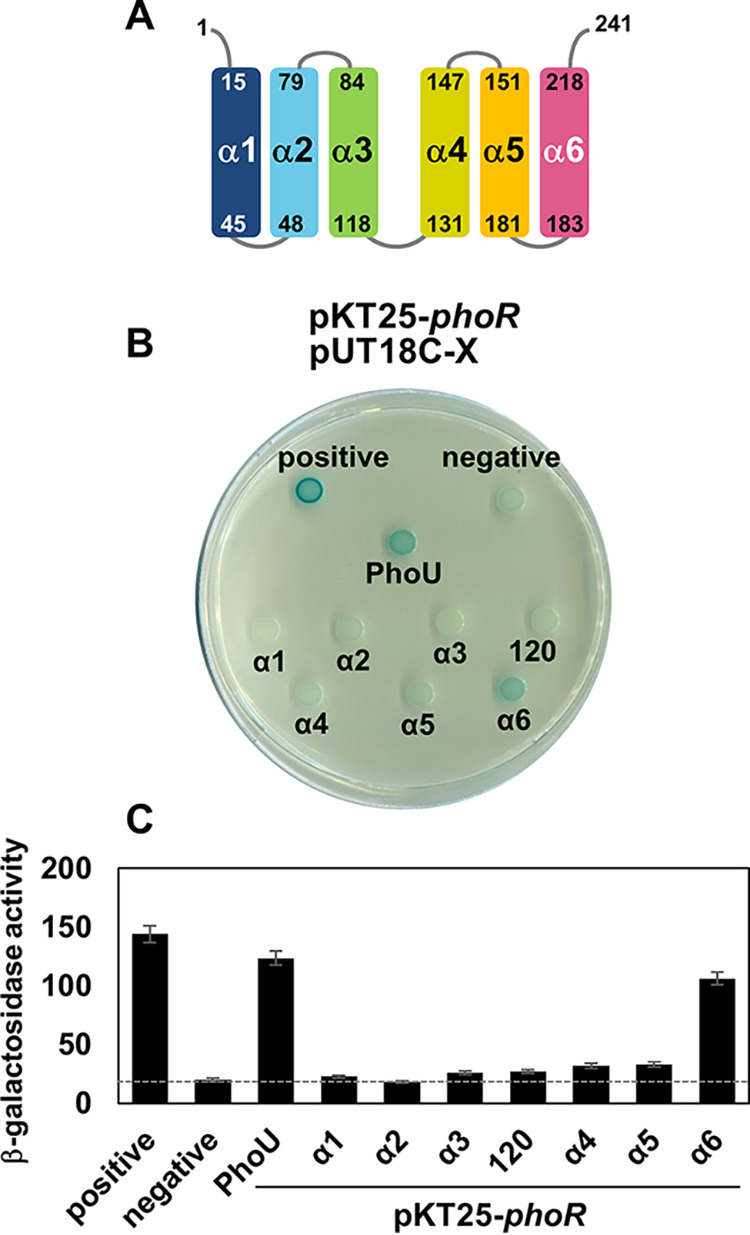
A region that includes α-helix 6 of PhoU is also required for PhoR interaction. (A) Schematic representation of the Salmonella PhoU protein. Numbers correspond to the positions of each helix in the full-length PhoU sequence (aa 1 to 241). (B) Bacterial two-hybrid assay between PhoR and PhoU or PhoU variant proteins. Escherichia coli BTH101 strains harboring two plasmids (pKT25 and pUT18C derivatives) expressing an N-terminal fusion of the *cyaA* T25 fragment to the coding region of the *phoR* gene and N-terminal fusions of the *cyaA* T18 fragment to the full-length *phoU* (PhoU), *phoU*_1–44_ (α1), *phoU*_1–77_ (α2), *phoU*_1–119_ (α3), *phoU*_1–147_ (α4), *phoU*_1–181_ (α5), *phoU*_1–218_ (α6), or *phoU*_1–120_ (PhoU120) genes or the pUT18C empty vector (negative) were spotted as indicated. Cells expressing both pUT18-*mgtC* and pKT25-*mgtR* are spotted as a positive control (37). Cells were spotted onto LB plates containing 80 μM X-Gal and 0.1 mM IPTG and incubated at 30°C for 40 h. Blue-colored colonies indicate a positive interaction. (C) β-Galactosidase assay from strains listed above. The average β-galactosidase activities (Miller units) are shown as mean ± SD (*n* = 3, independent measurements).

We then started to navigate which region(s) of the PhoU protein is required for PhoR interaction. Because PhoU consists of six α-helices (Fig. 2C), we created a series of T18-PhoU derivatives that were serially deleted from each α-helix from the C terminus (Fig. 3A). Only two constructs, T18 fused with the full-length PhoU (PhoU, amino acids [aa] 1 to 231) and a T18-PhoU derivative that harbors the coding region up to the end of α-helix 6 (α6, aa 1 to 218), retained the ability to interact with T25-PhoR (Fig. 3B and C). In contrast, all other T18-PhoU derivatives that harbor the coding regions up to α-helix 5 (α5, aa 1 to 181) or less (α1 to α4) lost the ability to interact with PhoR (Fig. 3B and C). These data suggest that a near full length of the PhoU protein is required for PhoR interaction, and the region containing and neighboring α-helix 6 (aa 182 to 218; Fig. S3A) is specifically required for this interaction.

10.1128/mbio.00811-22.3 FIG S3 Two additional regions of PhoU protein are involved in PhoR interaction. (A) Alignment of the amino acid sequences of the PhoU proteins from Salmonella enterica and Thermotoga maritima. Each helix (α1 to α6) of Salmonella is predicted based on the homology-based modeling using the crystal structure of Thermotoga maritima PhoU homolog 2 (PDB ID 1SUM). Six helices of the Salmonella PhoU protein are also presented as a diagram above the sequence. The gray line in the diagram represents the predicted region by the homology-based modeling (14 to 226). Sequences corresponding to α-helices of T. maritima are colored. Asterisks correspond to positions conserved in both species. (B) Schematic representation of the Salmonella PhoU protein. Numbers correspond to the positions of each helix in the full-length PhoU sequence (aa 1 to 241). Residues substituted in panel C are indicated as red dots. Residues substituted in Fig. 2 are indicated as blue dots. (C) Bacterial two-hybrid assay between PhoR and PhoU or PhoU variant proteins. Escherichia coli BTH101 strains harboring two plasmids, pUT18c and pKT25 derivatives, expressing an N-terminal fusion of the *cyaA* T25 fragment to the *phoR* gene and N-terminal fusions of the *cyaA* T18 fragment to the wild-type *phoU*, *phoU*^Q120A^, *phoU*^Q120G^, *phoU*^L124G^, *phoU*^L125A^, *phoU*^E181A^, *phoU*^E181G^, *phoU*^D182A^, *phoU*^D182G^, *phoU*^R184A^, and *phoU*^R184G^ genes, or the pUT18c empty vector (negative) were spotted as indicated. Cells expressing both pUT18-*mgtC* and pKT25-*mgtR* are spotted as a positive control (37). Cells were spotted onto LB plates containing 80 μM X-Gal and 0.1 mM IPTG and incubated at 30°C for 40 h. Blue-colored colonies indicate a positive interaction. (D) β-Galactosidase assay from strains listed above. The average β-galactosidase activities (Miller units) are shown as mean ± SD (*n* = 3, independent measurements). Download FIG S3, TIF file, 1.2 MB. Copyright © 2022 Choi et al. 2022 Choi et al. https://creativecommons.org/licenses/by/4.0/ This content is distributed under the terms of the Creative Commons Attribution 4.0 International license.

### Arg184 in the α-helix 6 of PhoU is required to interact with PhoR histidine kinase.

The region corresponding to amino acids 182 to 218 includes the α5-α6 loop and α-helix 6 itself (Fig. S3). We suspected that an additional interaction between PhoU and PhoR might occur near the α5-α6 loop or at the beginning of α-helix 6. This is because Ala147 and Arg148 in the α4-α5 loop are involved in PhoR interaction via the PAS domain (Fig. S3), and, based on structural modeling, it seems unlikely that α-helix 6 is involved in interacting with the same PAS domain, considering that most of α-helix 6 residues are far from the PhoU-PhoR PAS interface (Fig. S4A, top view).

10.1128/mbio.00811-22.4 FIG S4 There is a potential interaction between PhoU and the HK domain of the PhoR histidine kinase. (A) Top view of the PhoU protein. The Ala147 and Arg148 residues are indicated as a pink stick. (B) Homology-modeled structures of PhoR and PhoU. Download FIG S4, TIF file, 2.0 MB. Copyright © 2022 Choi et al. 2022 Choi et al. https://creativecommons.org/licenses/by/4.0/ This content is distributed under the terms of the Creative Commons Attribution 4.0 International license.

**FIG 4:**
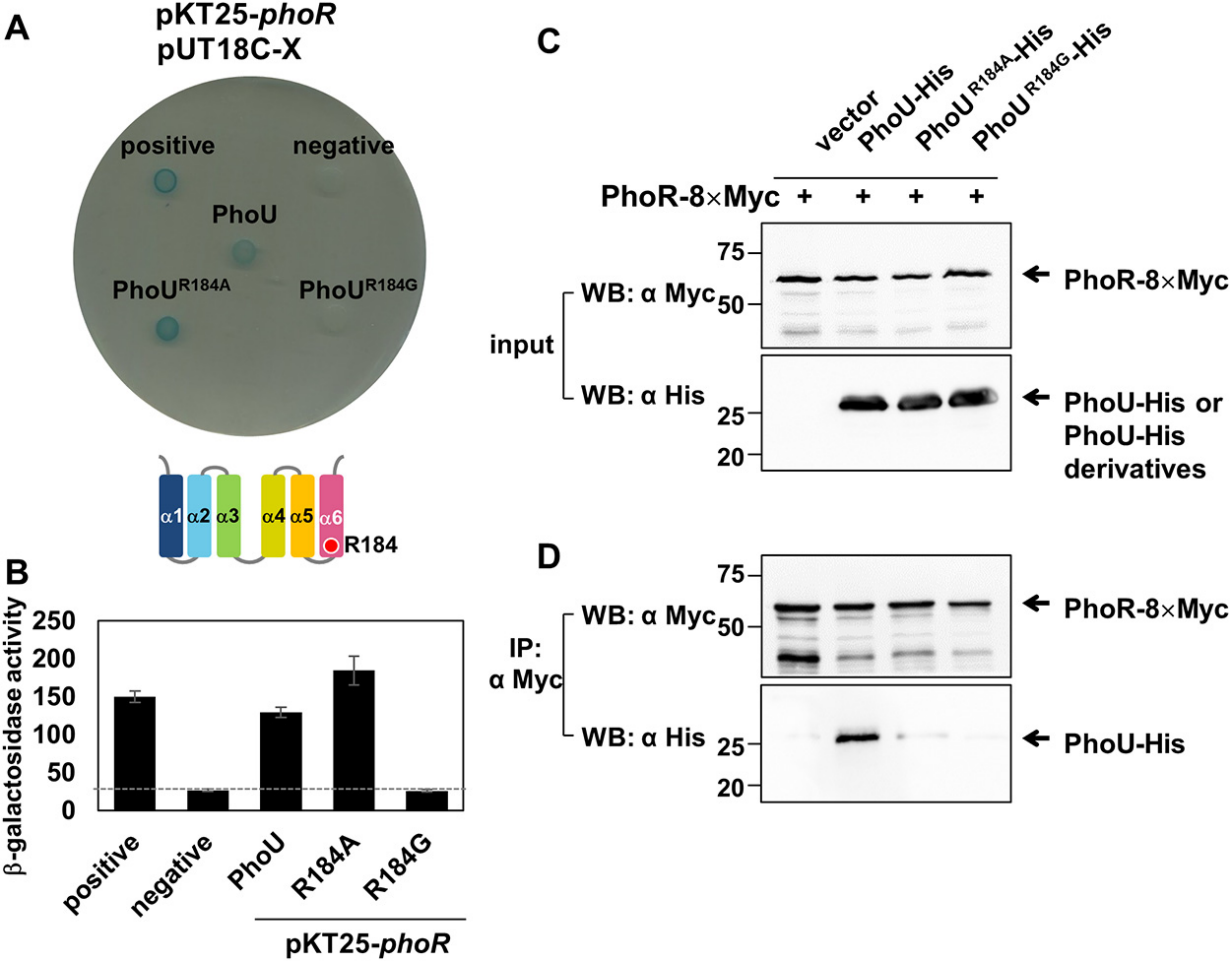
The PhoU Arg184 residue is required for PhoR interaction. (A) Bacterial two-hybrid assay between PhoR and PhoU or PhoU variant proteins. Escherichia coli BTH101 strains harboring two plasmids, pUT18c and pKT25 derivatives, expressing an N-terminal fusion of the *cyaA* T25 fragment to the *phoR* gene and N-terminal fusions of the *cyaA* T18 fragment to the wild-type *phoU*, *phoU*^R184A^, *phoU*^R184G^ genes, or the pUT18C empty vector (negative) were spotted as indicated. Cells expressing both pUT18-*mgtC* and pKT25-*mgtR* are spotted as a positive control (37). Cells were spotted onto LB plates containing 80 μM X-Gal and 0.1 mM IPTG and incubated at 30°C for 40 h. Blue-colored colonies indicate a positive interaction. (B) β-Galactosidase assay from strains listed above. The average β-galactosidase activities (Miller units) are shown as mean ± SD (*n* = 3, independent measurements). (C and D) The C-terminally 8×Myc-tagged PhoR protein immunoprecipitates PhoU-His. (C) Crude extracts prepared from Salmonella strains with the *phoR*-8×Myc gene expressing PhoU-His, PhoU^R184A^-His, PhoU^R184G^-His, or the empty vector (pBAD33) were detected with anti-Myc (top) and anti-His (bottom) antibodies. (D) Eluted fractions prepared from strains listed above were detected with anti-Myc (top) and anti-His (bottom) antibodies after immunoprecipitation with anti-Myc antibody-coated beads.

Based on our structural modeling, we selected Glu181, Asp182, and Arg184 residues to test for additional PhoR interactions (Fig. S3 and S4). As control experiments, we also selected Gln120 and Leu124 residues outside α-helix 6 because Gln120 and Leu124 residues are in the α3-α4 loop and are also in close proximity to those selected residues in the α5-α6 loop (Fig. S3). Among them, the Arg184 substitution to Gly completely lost the ability to interact with T25-PhoR (Fig. 4 and Fig. S3), indicating that the Arg184 residue in the α-helix 6 is required for PhoR interaction. We further demonstrated the requirement of the PhoU Arg184 residue for PhoR interaction by immunoprecipitation. The C-terminally Myc-tagged PhoR protein successfully immunoprecipitated the C-terminally His-tagged PhoU protein (Fig. 4). In contrast, the PhoR-GFP protein failed to immunoprecipitate a PhoU-His derivative with the Arg184-to-Ala or -Gly substitution (Fig. 4), supporting the requirement of Arg184 residue in PhoU-PhoR interaction. We could even recapitulate this interaction with the C-terminally green fluorescent protein (GFP)-tagged HK domain of PhoR even though the HK domain-PhoU interaction was much weaker than the interaction between full-length PhoR and PhoU (Fig. S2F and G). Please note that the Gln120-to-Ala substitution in PhoU also abolished the interaction between PhoU and PhoR (Fig. S3), but we did not follow the substitution further because the Gln120-to-Ala substitution also disrupted the interaction with the PstB ATPase, another PhoU-interacting protein in phosphate signaling (Fig. S5).

10.1128/mbio.00811-22.5 FIG S5 The *phoU* R184G substitution does not affect the interaction between PhoU and PstB. (A) Bacterial two-hybrid assay between PstB and PhoU or PhoU variant proteins. Escherichia coli BTH101 strains harboring two plasmids, pUT18c and pKT25 derivatives, expressing an N-terminal fusion of the *cyaA* T25 fragment to the *pstB* gene and N-terminal fusions of the *cyaA* T18 fragment to the wild-type *phoU*, *phoU*^Q120A^, *phoU*^Q120G^, *phoU*^R184A^, and *phoU*^R184G^ genes or the pUT18c empty vector (negative) were spotted as indicated. Cells expressing both pUT18-*mgtC* and pKT25-*mgtR* are spotted as a positive control (37). Cells were spotted onto LB plates containing 80 μM X-Gal and 0.1 mM IPTG and incubated at 30°C for 40 h. Blue-colored colonies indicate a positive interaction. (B) β-Galactosidase assay from strains listed above. The average β-galactosidase activities (Miller units) are shown as mean ± SD (*n* = 3, independent measurements). Download FIG S5, TIF file, 0.9 MB. Copyright © 2022 Choi et al. 2022 Choi et al. https://creativecommons.org/licenses/by/4.0/ This content is distributed under the terms of the Creative Commons Attribution 4.0 International license.

### The PhoU Arg184 residue is required for MgtC-mediated activation of *pho* genes but not low P_i_-mediated activation.

**FIG 5:**
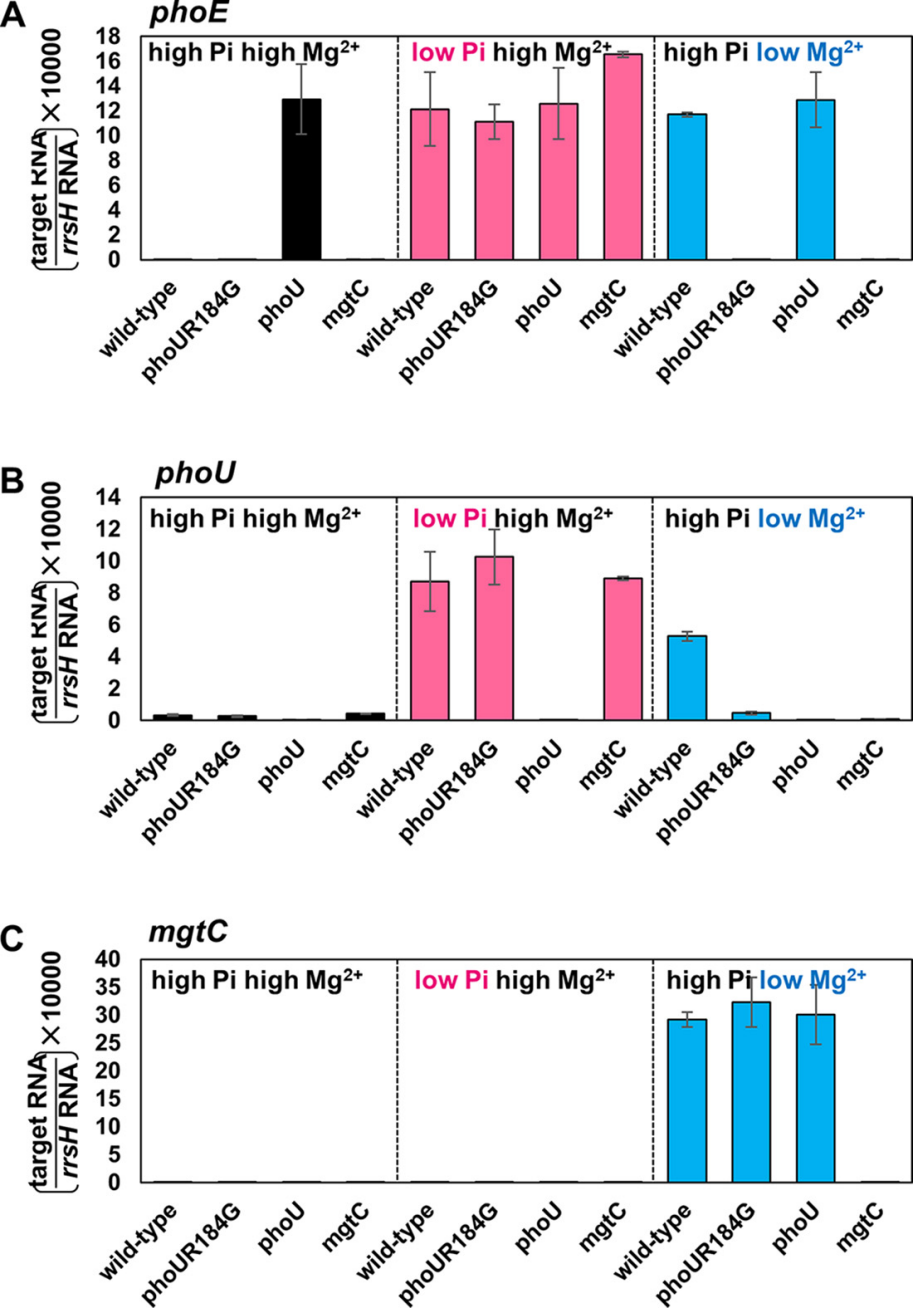
The *phoU* Arg184-to-Gly substitution prevents a low Mg^2+^-mediated increase in *phoE* mRNA levels but does not affect low P_i_-mediated *phoE* induction. (A to C) Relative mRNA levels of the *phoE* (A), *phoU* (B), and *mgtC* (C) genes in Salmonella strains with the wild-type *phoU* gene (14028s) or Arg184 to Gly-substituted *phoU* (SM235) genes and strains that deleted the *phoU* gene (SM101) or the *mgtC* gene (EL4). Bacteria were grown for 5 h in N-minimum media containing 10 mM Mg^2+^ and 10 mM P_i_ (high Mg^2+^, high P_i_), 10 mM Mg^2+^ and 0.01 mM P_i_ (high Mg^2+^, low P_i_), or 0.01 mM Mg^2+^ and 10 mM P_i_ (low Mg^2+^, high P_i_). Shown are the means ± SD (*n* = 3, independent measurements). Relative mRNA levels represent (target RNA/*rrsH* RNA) × 10,000.

Protein modeling suggests that the Arg184 residue is located on the opposite side of the Ala147 and Arg148 residues in the PhoU protein (Fig. 2B and Fig. S4). Given that the Ala147 and Arg148 residues in PhoU interact with PhoR possibly via the PAS domain, Arg184 appears to be in close proximity to the CA domain of the PhoR protein (Fig. S4). This configuration suggests that PhoU Arg184 might have contact with the CA domain in PhoR and also have a regulatory role in the PhoR histidine kinase-mediated signaling. To understand the physiological role of the PhoU-PhoR interaction via the PhoU Arg184 residue, we created a chromosomal mutant strain with the Arg184-to-Gly substitution in PhoU. We then tested the behaviors of the mutant in phosphate signaling. In the wild type, mRNA levels of the *phoE* gene were repressed in high-phosphate media but highly elevated in low-phosphate media (Fig. 5). Contrary to our expectation, the *phoU* Arg184-to-Gly substitution did not affect the expression patterns of *phoE* mRNA levels in either high- or low-phosphate media (Fig. 5). As a control experiment, *phoU* deletion derepressed *phoE* mRNA levels both in high- and low-phosphate media.

Because the *phoU* Arg184-to-Gly substitution had no effect on PhoB/PhoR-mediated phosphate signaling despite disrupting the PhoU-PhoR interaction, we tried to look for other conditions that could be affected by the *phoU* Arg184-to-Gly substitution. In a previous study, the Salmonella MgtC virulence factor also activates the PhoB-dependent genes independently of available phosphate concentration (16). MgtC activates PhoR histidine kinase by interacting with the CA domain (Fig. 1), which coincides with the potential contact site of PhoU Arg184 (Fig. 1 and Fig. S4). This suggests that the PhoU Arg184 residue might affect MgtC-mediated PhoR control. To test this, Salmonella strains with the wild-type *phoU* gene or the Arg184 to Gly-substituted *phoU* gene were grown in N-minimal medium with low Mg^2+^ to induce *mgtC* expression from the PhoP/PhoQ-dependent promoter (21) (Fig. 5C). As a control, the same strains were grown in N-minimal medium with high Mg^2+^ to repress *mgtC* expression. In the wild type, expression of the *phoE* gene was highly induced in low Mg^2+^ but repressed in high Mg^2+^ (Fig. 5), and the low Mg^2+^-mediated *phoE* induction is MgtC dependent because the *mgtC* deletion strain failed to increase in low Mg^2+^. The *phoU* Arg184-to-Gly substitution completely lost the ability to induce *phoE* expression, similar to that observed in the *mgtC* mutant (Fig. 5), indicating that PhoU is required for MgtC-mediated PhoR activation via the Arg184 residue. As a control, the *phoU* deletion mutant showed elevated *phoE* mRNA levels in all tested conditions (Fig. 5).

**FIG 6:**
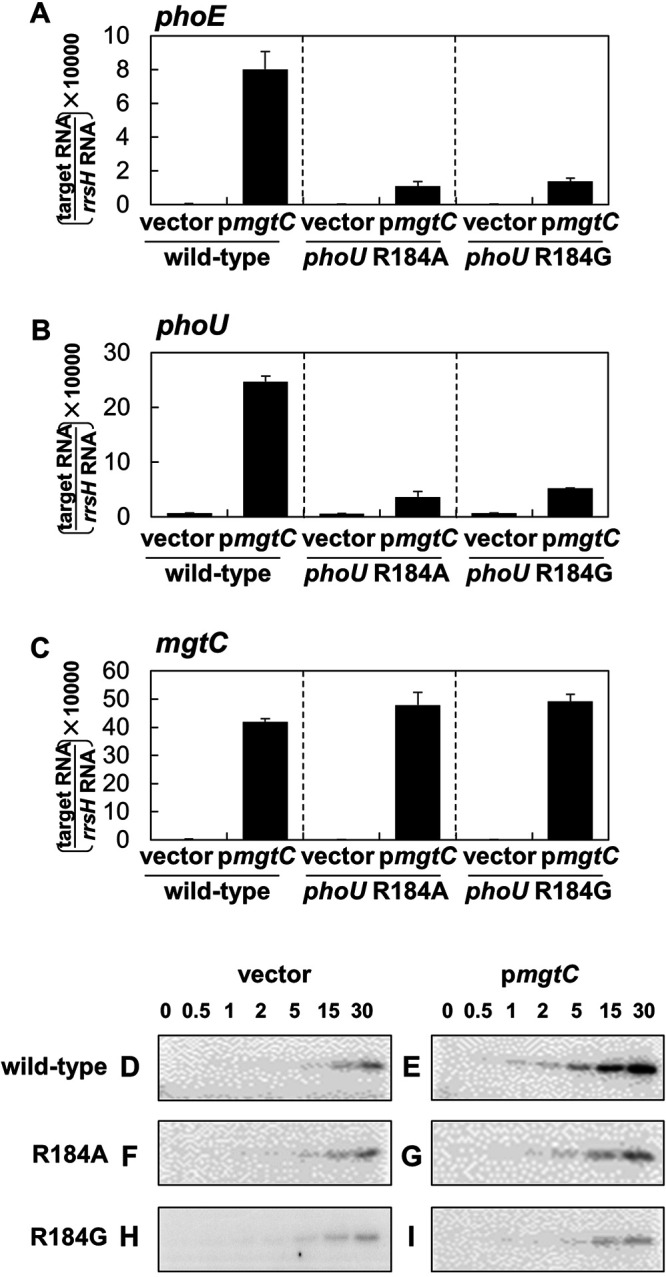
The *phoU* Arg184-to-Ala or -Gly substitution prevents an MgtC-mediated increase in *phoE* and *phoU* mRNA levels and PhoR autophosphorylation. (A to C) Relative mRNA levels of the *phoE* (A), *phoU* (B), and *mgtC* (C) genes in Salmonella strains with the wild-type *phoU* gene (14028s), Arg184 to Ala-substituted *phoU* (SM233), or Arg184 to Gly-substituted *phoU* (SM235) genes harboring a plasmid with the *mgtC* gene (p*mgtC*) or the vector (pUHE21-2lacI^q^). Bacteria were grown for 3 h in N-minimal medium containing 10 mM Mg^2+^ and then for an additional hour in the same medium containing 0.5 mM Mg^2+^ and 0.25 mM IPTG. Shown are the means ± SEM (*n* = 3, independent measurements). Relative mRNA levels represent (target RNA/*rrsH* RNA) × 10,000. (D to I) Autophosphorylation assay to determine the rate of PhoR phosphorylation. Levels of phospho-PhoR following incubation of membrane vesicles prepared from Salmonella strains with the wild-type *phoU* (14028s) (D and E), the Arg184 to Ala-substituted *phoU* (SM233) (F and G), or the Arg184 to Gly-substituted *phoU* genes (SM235) (H and I) expressing MgtC (D, F, and H) or the empty vector (E, G, and I) with [γ-^32^P]ATP at the indicated times (min). Bacteria were grown as described above in panels A to C, and the membrane vesicles were prepared as described in Materials and Methods.

This effect was further confirmed by introducing the *mgtC* gene heterologously expressed from an IPTG (isopropyl-β-d-thiogalactopyranoside)-inducible promoter. In the wild type, *mgtC* expression from an IPTG-inducible promoter increased *phoE* mRNA levels (Fig. 6). However, the chromosomal *phoU* mutant strains with Arg184-to-Ala or -Gly substitutions exhibited reduced *phoE* mRNA levels despite high levels of *mgtC* expression (Fig. 6), indicating that PhoU Arg184 is required for MgtC-mediated *phoE* expression.

**FIG 7:**
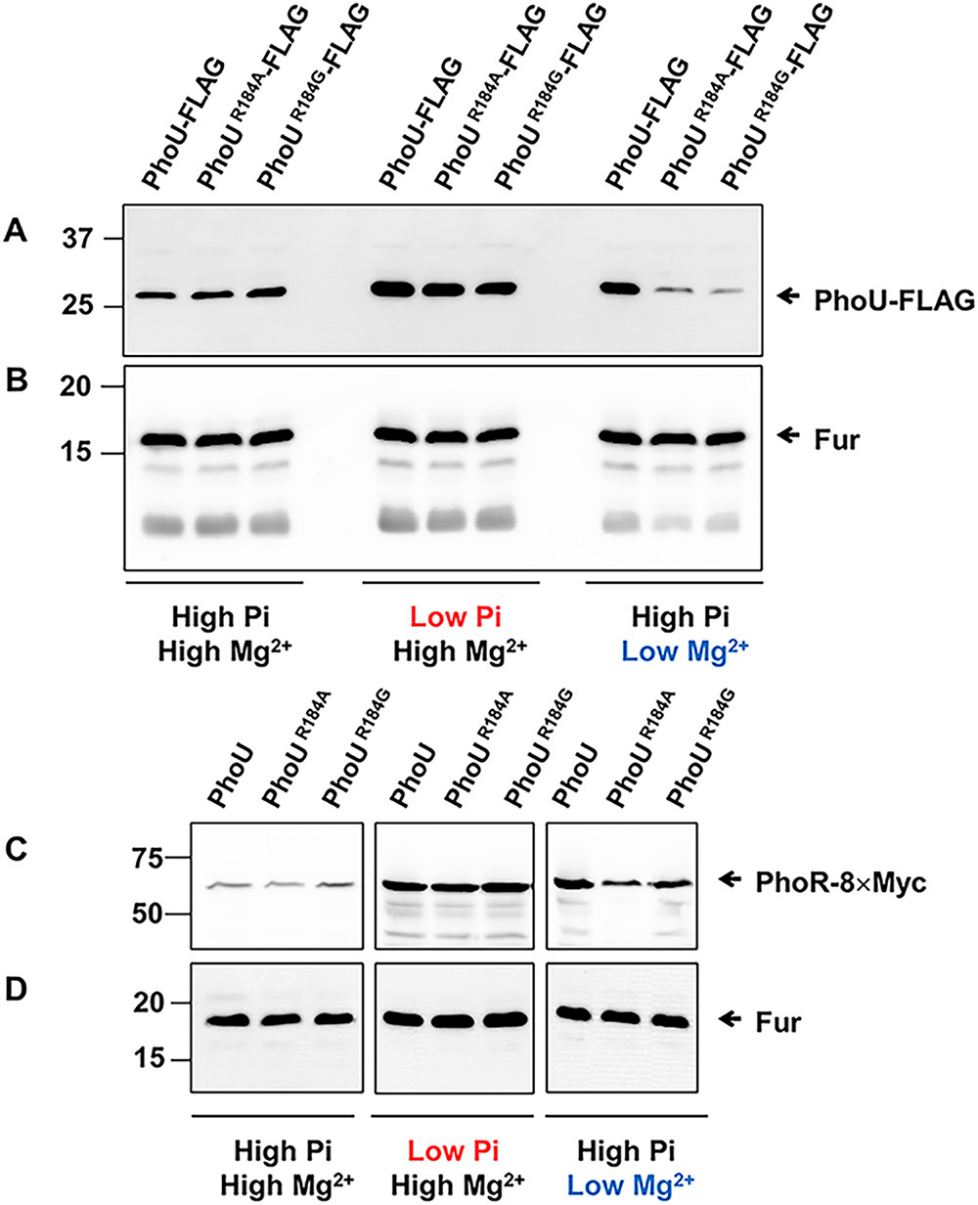
The *phoU* Arg184-to-Ala or -Gly substitutions decrease PhoU and PhoR protein levels in low Mg^2+^ but not in low P_i_. (A and B) Western blot analysis of crude extracts prepared from strains with the wild-type *phoU*-FLAG gene (SM458) or *phoU*-FLAG derivatives with the Arg184 codon substituted by Ala (SM459) or Gly (SM460) codons. Blots were probed with anti-FLAG (A) or anti-Fur (B) antibodies to detect PhoU-FLAG and Fur proteins, respectively. (C and D) Western blot analysis of crude extracts prepared from strains with the wild-type *phoU* gene (SM454) or *phoU* derivatives with the Arg184 codon substituted by Ala (SM455) or Gly (SM456) codons in the chromosomal *phoR*-8×Myc background. Blots were probed with anti-Myc (A) or anti-Fur (B) antibodies to detect PhoR-8×Myc and Fur proteins, respectively. Bacteria were grown for 5 h in N-minimal media containing combinations of 10 mM (high) or 0.01 mM (low) P_i_ and 10 mM (high) or 0.01 mM (low) Mg^2+^.

Given that both PhoU and PhoR histidine kinase belong to PhoB/PhoB-controlled genes, we created strains with the C-terminally FLAG-tagged *phoU* gene or C-terminally 8×Myc tagged *phoR* gene at its chromosomal locations and measured PhoU or PhoR protein levels in the presence of the *phoU* Arg184-to-Ala or -Gly substitutions. In low P_i_, both PhoU and PhoR protein levels increased compared to high P_i_ and were unaffected by the *phoU* Arg184-to-Ala or -Gly substitutions (Fig. 7). However, similar to what we detected in *phoE* mRNA levels, the *phoU* Arg184-to-Ala or -Gly substitutions exhibited a defect in low Mg^2+^-mediated induction of PhoU and PhoR protein levels (Fig. 7), supporting a notion that PhoU Arg184 residue is required for MgtC-mediated PhoR activation, possibly via interacting with the HK domain of PhoR.

Next, we determined epistasis between the *phoU* Arg184-to-Gly and the above-mentioned *phoU* Ala147-to-Glu substitutions. To test this, we created a strain where both Ala147 and Arg184 codons in the *phoU* gene were substituted by Glu and Gly codons, respectively. Then, we investigated the expression behavior of the A147E- and R184G-substituted *phoU* mutant in low phosphate or low Mg^2+^. The expression patterns of the A147E- and R184G-substituted *phoU* mutant were identical to those of the *phoU* A147E mutant in all tested conditions (Fig. S6), indicating that the *phoU* Ala147-to-Glu substitution is dominant over the *phoU* Arg184-to-Gly substitution.

10.1128/mbio.00811-22.6 FIG S6 The *phoU* Ala147-to-Glu substitution is dominant over the Arg184-to-Gly substitution. (A to C) Relative mRNA levels of the *phoE* (A), *phoU* (B), and *mgtC* (C) genes in Salmonella strains with the wild-type *phoU* gene (14028s), Ala147 to Glu-substituted *phoU* (SM323), Arg184 to Gly-substituted *phoU* (SM235), or both A147E and R184G-substituted *phoU* (SM427) genes. Bacteria were grown for 5 h in N-minimal medium containing 10 mM Mg^2+^ and 10 mM P_i_ (high Mg^2+^, high P_i_), 10 mM Mg^2+^ and 0.01 mM P_i_ (high Mg^2+^, low P_i_), or 0.01 mM Mg^2+^ and 10 mM P_i_ (low Mg^2+^, high P_i_). Shown are the means ± SD (*n* = 3, independent measurements). Relative mRNA levels represent (target RNA/*rrsH* RNA) × 10,000. Download FIG S6, TIF file, 0.9 MB. Copyright © 2022 Choi et al. 2022 Choi et al. https://creativecommons.org/licenses/by/4.0/ This content is distributed under the terms of the Creative Commons Attribution 4.0 International license.

### The PhoU Arg184 residue is required for MgtC-mediated PhoR autophosphorylation.

MgtC binds to the Leu421 residue of the PhoR CA domain and promotes autophosphorylation, thus increasing expression of PhoB-dependent genes (16). Because the PhoU Arg184 residue is required for the MgtC-mediated increase in *phoE* mRNA levels, we wondered whether the PhoU Arg184 residue is also involved in PhoR autophosphorylation when MgtC is present. We grew cells in low Mg^2+^ to increase MgtC production and isolated membrane fractions to measure PhoR autophosphorylation. When membrane fractions isolated from wild-type cells were incubated with γ-radiolabeled ATP, phosphorylated PhoR proteins were detected from 30 s and strongly increased up to 30 min (Fig. S7). However, the *phoU* Arg184-to-Gly substitution delayed the appearance of phosphorylated PhoR and also exhibited low levels of phospho-PhoR (Fig. S7), suggesting that the PhoU Arg184 residue is involved in MgtC-mediated PhoR autophosphorylation. As a control, the *phoU* Arg184-to-Gly substitution did not affect the behavior of PhoR autophosphorylation when grown in low phosphate (Fig. S7), and the levels of phospho-PhoR of both strains were low in a PhoB- and PhoP-repressing (10 mM P_i_ and 10 mM Mg^2+^) condition (Fig. S7).

10.1128/mbio.00811-22.7 FIG S7 *phoU* Arg184-to-Gly substitution decreases low Mg^2+^-mediated PhoR autophosphorylation but not low P_i_-mediated PhoR autophosphorylation. (A to F) Autophosphorylation assay to determine the rate of PhoR phosphorylation. Levels of PhoR/PhoP following incubation of membrane vesicles prepared from Salmonella strains with the wild-type *phoU* (14028s; A to C) or the Arg184 to Gly-substituted *phoU* gene (SM235; D to F) with [γ-^32^P]ATP at the indicated times (min). Bacteria were grown for 5 h in N-minimal medium containing 0.01 mM Mg^2+^ and 10 mM P_i_ (low Mg^2+^ high P_i_) (A and D), 10 mM Mg^2+^ and 0.01 mM P_i_ (high Mg^2+^ low P_i_) (B and E), or 10 mM Mg^2+^ and 10 mM P_i_ (high Mg^2+^, high P_i_) (C and F) and membrane vesicles were prepared as described in Materials and Methods. Download FIG S7, TIF file, 0.7 MB. Copyright © 2022 Choi et al. 2022 Choi et al. https://creativecommons.org/licenses/by/4.0/ This content is distributed under the terms of the Creative Commons Attribution 4.0 International license.

Because the *phoU* Arg184-to-Gly substitution decreased PhoU and PhoR protein levels in low Mg^2+^ due to its autoregulation (Fig. 7), the autophosphorylation defect of the *phoU* Arg184-to-Gly mutant in low Mg^2+^ might be due to low levels of PhoU and PhoR proteins. To avoid this, we also measured autophosphorylation of Salmonella strains that were grown in high Mg^2+^ to produce similar levels of PhoR and PhoU proteins (Fig. 7) and expressing MgtC protein from a plasmid. Consistently to what we detected in *phoE* and *phoU* mRNA levels, wild-type Salmonella expressing MgtC protein from the plasmid increased the rate of PhoR autophosphorylation compared to those expressing the empty vector (Fig. 6D and E). However, MgtC expression from the *phoU* Arg184-to-Ala or -Gly substitution mutants did not affect the rate of PhoR autophosphorylation (Fig. 6G and I), indicating that the PhoU Arg184 residue is indeed required for MgtC-mediated PhoR autophosphorylation.

### The PhoU Arg184 residue is required for Salmonella virulence.

**FIG 8:**
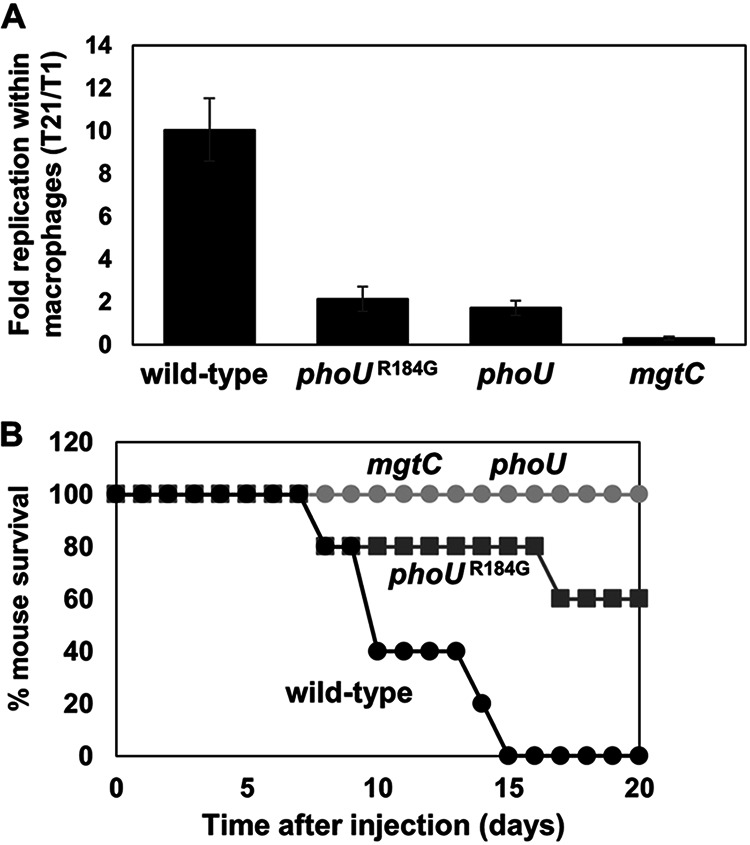
PhoU Arg184-mediated PhoR autophosphorylation is required for Salmonella virulence. (A) Arg184-to-Gly substitution in PhoU decreases survival inside macrophages. Survival inside J774A.1 macrophage cells of the wild-type Salmonella (14028s), the *phoU* chromosomal mutant with Arg184 replaced by the Gly codon (SM235), the *phoU* deletion mutant (SM101), and the *mgtC* deletion mutant (EL4) at 21 h postinfection (T21). Fold replication represents the number of bacteria at T21/number of bacteria at T1. Shown are the means and SD from three independent infections. (B) Arg184-to-Gly substitution in PhoU decreases mouse virulence. Survival of C3H/HeN mice inoculated intraperitoneally with ~10^3^ CFU of the Salmonella strains listed above.

Given that the *phoU* Arg184-to-Gly substitution affects MgtC-mediated *phoE* expression (Fig. 5) and that the *mgtC* gene is required for intramacrophage survival (22, 23) (Fig. 8A), we wondered whether PhoU Arg184-mediated PhoR autophosphorylation is required for Salmonella survival inside macrophages. When we infected Salmonella into the macrophage-like cell line J774A.1, the *phoU* variant with the Arg184-to-Gly substitution decreased replication within macrophages, reaching only 20% of that of the wild type (Fig. 8A). This is almost as defective as a mutant strain with the *phoU* deletion, suggesting that the Arg184 residue largely contributes to PhoU’s role in Salmonella pathogenesis. Similarly, when we injected ~3,000 CFU of Salmonella strains intraperitoneally into C3H/HeN mice, the Salmonella strain with the *phoU* Arg184-to-Gly substitution attenuated mouse virulence compared to the wild type (Fig. 8B). As controls, strains that deleted either the *phoU* or *mgtC* genes were completely defective in mouse virulence. Collectively, these data indicate that the PhoU Arg184 residue is involved in Salmonella virulence, possibly by controlling the expression of *pho* regulon genes via PhoR autophosphorylation.

## DISCUSSION

Using on model-based substitutions, we determined that the PhoU regulator binds to PhoR histidine kinase via the HK domain. Because the interaction between PhoU and the PAS domain of PhoR histidine kinase was already established in E. coli (5), this suggests that PhoU could interact with PhoR histidine kinase in at least two different sites, the PAS domain and the CA domain (Fig. 1). Substitution of Ala147 to Glu in the Salmonella PhoU protein, which likely prevents PhoU-PhoR PAS domain interaction, derepressed *phoE* mRNA levels in high phosphate, in agreement with a previous finding in E. coli that PhoU functions as a negative regulator via interaction with the PhoR PAS domain (5). Constitutively elevated *phoE* expression in the *phoU* Ala147-to-Glu substitution suggests that PhoU’s binding to the PhoR PAS domain suppresses autophosphorylation activity, probably by inducing a conformation change. However, repression of *phoE* expression could also be achieved by either preventing phosphate transfer from PhoR to PhoB or stabilizing phosphatase activity toward phosphorylated PhoB protein. Given that the phosphatase activity and the transition between kinase and phosphatase activities of Salmonella PhoR histidine kinase have not been carefully examined yet, it needs to be investigated to understand a mechanism underlying PhoU-mediated inhibition of PhoR activity.

Interestingly, the introduction of the *phoU* Arg184-to-Gly substitution that disrupts the interaction between PhoU and PhoR HK domain did not affect *phoE* mRNA levels in high- or low-phosphate conditions (Fig. 5), suggesting that the interaction between the PhoU-PhoR HK domain is not involved in repressing expression of *pho* regulon in high phosphate. Instead, PhoU’s interaction with the PhoR HK domain seems to be required for inducing expression of the *pho* regulon when Salmonella is within macrophages because the *phoU* Arg184-to-Gly substitution failed to induce *phoE* mRNA levels under the conditions when the MgtC virulence factor was highly expressed (Fig. 5 and 6). Given that the PhoU protein exerts its opposing effects on expression of the *pho* regulon by interacting with PhoR histidine kinase at two different sites, PhoU should be considered a modulator/molecular adaptor of *pho* expression rather than simply a negative regulator. By creating amino acid substitutions instead of the *phoU* deletion, we could dissect PhoU’s regulatory roles in repressing *pho* expression in high phosphate and in activating *pho* expression in the presence of the MgtC virulence factor that is highly expressed in low Mg^2+^ or during Salmonella infection (24).

PhoU's function as a molecular adaptor was also described in a previous report (13). In E. coli, when both phosphate and potassium are limiting, the phosphate-sensing PhoB/PhoR two-component system also controls a counterion signaling pathway via the potassium-sensing KdpE/KdpD two-component system (13). In this case, PhoU seems to connect these two regulatory pathways given that PhoU interacts with both KdpD and PhoR histidine kinases in the bacterial two-hybrid assay and *phoU* deletion derepresses KdpE-dependent expression of KdpFABC K^+^ transporter independent of K^+^ concentration in the *kdpD* deletion background. However, its physiological relevance remains unclear, given that the effect of PhoU on potassium signaling can be clearly seen in a strain lacking the KdpD sensor kinase (13). Similar to PhoU, nonphosphorylated EIIA^Ntr^, a nitrogen PTS component, is also involved in K^+^ homeostasis in E. coli via protein-protein interaction. However, instead of connecting two regulatory pathways, monophosphorylated EIIA^Ntr^ controls K^+^ homeostasis differently depending on available K^+^ concentration. In low K^+^, EIIA^Ntr^ binds to KdpD histidine kinase and activates KdpE-dependent expression of KdpFABC high-affinity K^+^ transporter (25). In high K^+^, EIIA^Ntr^ directly binds to TrkA, a peripheral membrane protein of Trk low-affinity K^+^ transporter, and inhibits its activity (26), which is a major K^+^ transporter at high K^+^ conditions.

Previously, the MgtC virulence protein activated autophosphorylation of PhoR histidine kinase by directly binding to the PhoR CA domain and promoted expression of *pho* genes (16). The Leu421 residue in the PhoR CA domain was determined as a key residue required for this interaction. Because MgtC stimulates PhoR autophosphorylation independently of available phosphate concentration, it was unclear whether PhoU still binds to PhoR or participates in MgtC-mediated activation of PhoR histidine kinase. The fact that the PhoU Arg184 residue is also required for interacting with the PhoR HK domain (possibly via the CA domain) and stimulating MgtC-mediated PhoR autophosphorylation led us to speculate that the Salmonella MgtC virulence protein might activate *pho* expression via three molecular interactions between MgtC, PhoU, and PhoR histidine kinase. However, curiously enough, MgtC did not directly interact with PhoU in a previous two-hybrid study (16), despite both PhoR and PhoU proteins being required for MgtC-mediated activation of PhoR autophosphorylation. Thus, it seems that PhoR histidine kinase makes contact with both MgtC and PhoU proteins via its CA domain but accommodates these proteins at different surface areas, which needs further attention to understand how these molecular interactions occur. Finally, by creating substitution mutants that enable us to address a detailed function, we uncovered an unexpected role of PhoU, which was previously hidden by an effect of gene deletion. This finding will allow us to understand a detailed molecular mechanism of phosphate signaling in bacteria.

## MATERIALS AND METHODS

### Bacterial strains, plasmids, oligodeoxynucleotides, and growth conditions.

Bacterial strains and plasmids used in this study are listed in Table S1 in the supplemental material. All S. enterica serovar Typhimurium strains were derived from the wild-type strain 14028s (27) and were constructed by the one-step gene inactivation method (28) and/or P22-mediated transduction as previously described (29). DNA oligonucleotides are listed in Table S1. Bacteria were grown at 37°C in Luria-Bertani (LB) broth and N-minimal media (30) supplemented with 0.1% Casamino Acids, 38 mM glycerol, and the indicated concentrations of MgCl_2_. For low-phosphate N-minimal media, 10 mM KH_2_PO_4_ in the N-minimal medium was replaced by 0.01 mM KH_2_PO_4_. E. coli DH5α was used as the host for preparing plasmid DNA, and BTH101 lacking the *cya* gene was used as the host for the bacterial two-hybrid system (31). Ampicillin was used at 50 μg mL^−1^, chloramphenicol at 20 μg mL^−1^, kanamycin at 20 μg mL^−1^, tetracycline at 10 μg mL^−1^, and fusaric acid (32) at 12 μg mL^−1^. IPTG (isopropyl β-D-1-thiogalactopyranoside) was used at 0.25 mM, l-arabinose at 0.2% (wt/vol), and X-Gal (5-bromo-4-chloro-3-indolyl-β-d-galactopyranoside) at 80 μM.

10.1128/mbio.00811-22.8 TABLE S1 Bacterial strains, plasmids, and oligonucleotides used in this study. Download Table S1, DOCX file, 0.07 MB. Copyright © 2022 Choi et al. 2022 Choi et al. https://creativecommons.org/licenses/by/4.0/ This content is distributed under the terms of the Creative Commons Attribution 4.0 International license.

### Bacterial two-hybrid assay.

To assess protein-protein interactions *in vivo*, a bacterial two-hybrid (BACTH) assay was conducted as described (31). The Escherichia coli strain BTH101 lacking the *cya* adenylate cyclase gene was cotransformed with derivatives of the pUT18, pUT18c, and pKT25 plasmids. The strains were grown overnight at 37°C in LB supplemented with ampicillin (50 μg mL^−1^) and kanamycin (50 μg mL^−1^). Then, 4 μL of cells were spotted on solid LB medium with 500 μM IPTG, 100 μM ampicillin, 100 μM kanamycin, and 80 μM X-Gal, followed by incubation for 40 h or 60 h at 30°C. (For quantitative analysis, β-galactosidase assays were performed [33].)

### Protein structure modeling and protein docking modeling.

We used Protein Homology/analogy Recognition Engine v2.0 (Phyre2) (34) to model the structures of the cytoplasmic portion of the PhoR protein (corresponding to amino acids 52 to 431) and the full-length of the PhoU protein from Salmonella enterica serovar Typhimurium 14028s. The structure of the PhoR protein was modeled based on the structure of VicK from Streptococcus mutans (PDB ID 4I5S), and the structure of the PhoU protein was modeled based on the structure of the PhoU homolog 2 from Thermotoga maritima (PDB ID 1SUM) or PhoU from Pseudomonas aeruginosa (PDB ID 4Q25). Then, we used the ClusPro web server to investigate a potential interaction between Salmonella PhoR and PhoU proteins (35).

### Immunoprecipitation assay.

The interaction between the PhoU and PhoR proteins was investigated in wild-type Salmonella expressing the *phoU* gene or its derivatives from an arabinose-inducible promoter (pBAD33-*phoU*-His, pBAD33-*phoU*^Arg184Ala^-His, or pBAD33-*phoU*^Arg184Gly^-His) in the *phoR-*8×Myc background (*phoR-*8×Myc) (36). Cells were grown overnight in N-minimal medium containing 10 mM Mg^2+^. A 1:100 dilution of the overnight grown bacterial culture was inoculated in 15 mL of N-minimal medium containing 10 mM Mg^2+^ and grown for 3 h. Cells were then washed and transferred to 15 mL of N-minimal medium containing 0.5 mM Mg^2+^ and 1 mM l-arabinose and grown for 1 h. Cells were normalized by measuring the optical density at 600 nm (OD_600_). Crude extracts were prepared in Tris-buffered saline (TBS) buffer by sonication. For a pulldown assay with anti-GFP antibodies, 50 μL of the crude extracts were kept for input, and 400 μL of the protein extracts were mixed with 2 μL of anti-Myc (MBL Life Science) and 40 μL of protein G Sepharose 4 Fast Flow (Cytiva; catalog no. 17-0618-01) for 2 h at 4°C on a nutator (Benchmark Scientific). Beads were washed with TBS washing buffer three times, and then the bound proteins were eluted in SDS sample buffer. The eluates were resolved on 12% SDS-polyacrylamide gels, transferred to nitrocellulose membrane, and analyzed by Western blotting using anti-His (1:20,000 dilution; Rockland; catalog no. 600-401-382) and anti-Myc (1:3,000 dilution; MBL Life Science; catalog no. M192-3) antibodies overnight. The blots were washed and hybridized with anti-mouse IgG horseradish peroxidase-linked whole antibody (1:10,000 dilution; Amersham; catalog no. NA931) for 1 h and detected using SuperSignal West Femto maximum sensitivity substrate (Thermo Fisher). To investigate the interaction between PhoU and the HK domain of the PhoR protein, Salmonella strains with the C-terminally FLAG-tagged *phoU* gene and its derivatives expressing the C-terminally *gfp*-tagged HK domain of the *phoR* gene (pTGFP-*phoR*^HK^) or the empty vector (pTGFP) were used, and an immunoprecipitation assay was performed. The pulled-down PhoR^HK^-GFP proteins and the immunoprecipitated PhoU-FLAG proteins were detected with anti-GFP (1:1,000 dilution; Roche; catalog no. 11814460001) and anti-FLAG (1:3,000 dilution; Millipore; catalog no. F7425) antibodies, respectively. For secondary antibody, anti-mouse IgG horseradish peroxidase (HRP)-linked whole antibody (1:10,000 dilution; Amersham; catalog no. NA931) and anti-rabbit IgG horseradish peroxidase-linked antibody (1:10,000 dilution; Thermo Fisher; catalog no. 31460) were used to detect PhoR^HK^-GFP and PhoU-FLAG proteins, respectively.

### Membrane vesicle preparation.

Cells were for 5 h in N-minimal media containing combinations of 10 mM (high) or 0.01 mM (low) Mg^2+^ and 10 mM (high) or 0.01 mM (low) P_i_. Cells were normalized by measuring OD_600_. Crude extracts were prepared in TBS buffer by sonication and centrifuged supernatants for 2 h at 40,000 × *g* (Optima TLX ultracentrifuge, type 90 Ti rotor; Beckman Coulter). The pellets were resuspended in 50 μL TBS buffer. The protein concentration in the prepared membrane fractions was determined using a NanoDrop machine (Thermo Fisher).

### Measuring autophosphorylation of PhoR histidine kinase.

As previously described (16), 50 μL of membrane vesicles expressing wild-type PhoU or PhoU Arg184Gly were incubated in 100 μL of TBS containing 1 mM MgCl_2_ at room temperature. The reaction was started with the addition of [γ-^32^P]ATP (10 μCi; PerkinElmer) to the mixture. A 10-μL aliquot was taken at different time points and mixed with 10 μL of 5× SDS loading buffer (Biosesang) to stop the reaction. The samples were kept on ice until they were loaded onto a 12% SDS-polyacrylamide gel. After electrophoresis, the gel was dried on Whatman filter paper using model 583 gel dryer (Bio-Rad), and the phosphorylated PhoR proteins were visualized by a phosphoimaging on a Typhoon scanner (GE Healthcare). The phosphorylated PhoR proteins were identified using samples prepared from wild-type and the *phoR* mutant Salmonella grown for 5 h in N-minimal medium containing 0.01 mM Pi, a PhoB/PhoR-inducing condition.

### Quantitative real-time PCR.

Total RNA was isolated using RNeasy kit (Qiagen) according to the manufacturer’s instructions. The purified RNA was quantified using a NanoDrop machine (Thermo Fisher). cDNA was synthesized using PrimeScript RT reagent kit (TaKaRa). The mRNA levels of the *mgtC* and *phoE* genes were measured by quantifying the cDNA using SYBR green PCR master mix (Toyobo) and the appropriate primers (7530/7531 for the *mgtC* gene, KHQ015/KHQ016 for the *phoE* gene, and KHQ097/KHQ098 for the *phoU* gene) and monitored using a StepOnePlus real-time PCR system (Applied Biosystems). The mRNA levels of each target gene were calculated using a standard curve of 14028s genomic DNA with known concentration, and data were normalized to the levels of 16S rRNA amplified with primers 6970 and 6971.

### Western blot analysis.

Cells were grown for 5 h in 15 mL of N-minimal medium containing combinations of 10 mM or 0.01 mM Mg^2+^ and 10 mM or 0.01 mM P_i_. Cells were normalized by measuring the OD_600_. Crude extracts were prepared in TBS by sonication. Whole-cell lysates were resolved on 12% SDS-polyacrylamide gels and were transferred onto nitrocellulose membranes. The blots were incubated with monoclonal anti-FLAG antibodies (1:3,000 dilution; Millipore; catalog no. F7425) and anti-Myc antibodies (1:3,000 dilution; MBL Life Science; catalog no. M192-3) overnight to detect PhoU-FLAG and PhoR-8×Myc proteins, respectively. The blots were developed by incubation with anti-rabbit IgG horseradish peroxidase-linked antibody (Ab) (1:10,000 dilution; Thermo Fisher; catalog no. 31460) and anti-mouse IgG HRP-linked whole Ab (1:10,000 dilution; Amersham; catalog no. NA931) for 1 h and were visualized using SuperSignal West Femto maximum-sensitivity substrate (Thermo Fisher).

### Macrophage survival assay.

Intramacrophage survival assays were conducted using the macrophage-like cell line J774 A.1. Briefly, 5 × 10^5^ macrophages in Dulbecco’s modified Eagle’s medium (DMEM) supplemented with 10% fetal bovine serum (FBS) were seeded in 24-well plates and cultured at 37°C. Overnight grown bacteria were added to the macrophages at a multiplicity of infection (MOI) of 10. The plates were centrifuged at 1,000 rpm for 5 min at room temperature and incubated for an additional 20 min. Then, the extracellular bacteria were washed three times with phosphate-buffered saline (PBS) and killed by incubation with DMEM supplemented with 10% FBS and 120 μg mL^−1^ gentamicin for 1 h. For measuring the number of bacteria at 1 h, cells were lysed with PBS containing 0.1% Triton X-100 and plated on Luria-Bertani (LB) plates with appropriate dilutions. For measuring the number of bacteria at 21 h, the DMEM was replaced after 1 h with fresh DMEM containing 12 μg mL^−1^ gentamicin, and the incubation was continued at 37°C. After 21 h, cells were lysed with PBS containing 0.1% Triton X-100 and plated on LB plates. The percentage survival was obtained by dividing the number of bacteria recovered after 21 h by the number of bacteria recovered at 1 h. All experiments were performed in triplicate, and the results are representative of at least three independent experiments.

### Mouse virulence assay.

Six- to 8-week-old female C3H/HeN mice were inoculated intraperitoneally with ~10^3^ CFU of Salmonella strains. Mouse survival was followed for 21 days. Virulence assays were conducted three times with similar outcomes, and the data correspond to groups of five mice. All animals were housed in a temperature- and humidity-controlled room, in which a 12-h light/12-h dark cycle was maintained. All procedures were performed according to the protocols (KW-181010-1) approved by the Institutional Animal Care and Use Committee of the Kangwon National University.

### Data availability.

All other relevant data are available from the corresponding author upon reasonable request.

10.1128/mbio.00811-22.9 TEXT S1 Supplemental materials and methods. Download Text S1, DOCX file, 0.04 MB. Copyright © 2022 Choi et al. 2022 Choi et al. https://creativecommons.org/licenses/by/4.0/ This content is distributed under the terms of the Creative Commons Attribution 4.0 International license.
