# Supplementary figures and images for: A Dual Regulatory Role of the PhoU Protein in Salmonella Typhimurium
Source: mBio. 2022 May 31;13(3):e00811-22. doi: 10.1128/mbio.00811-22 (PMC9239213; doi:10.1128/mbio.00811-22)

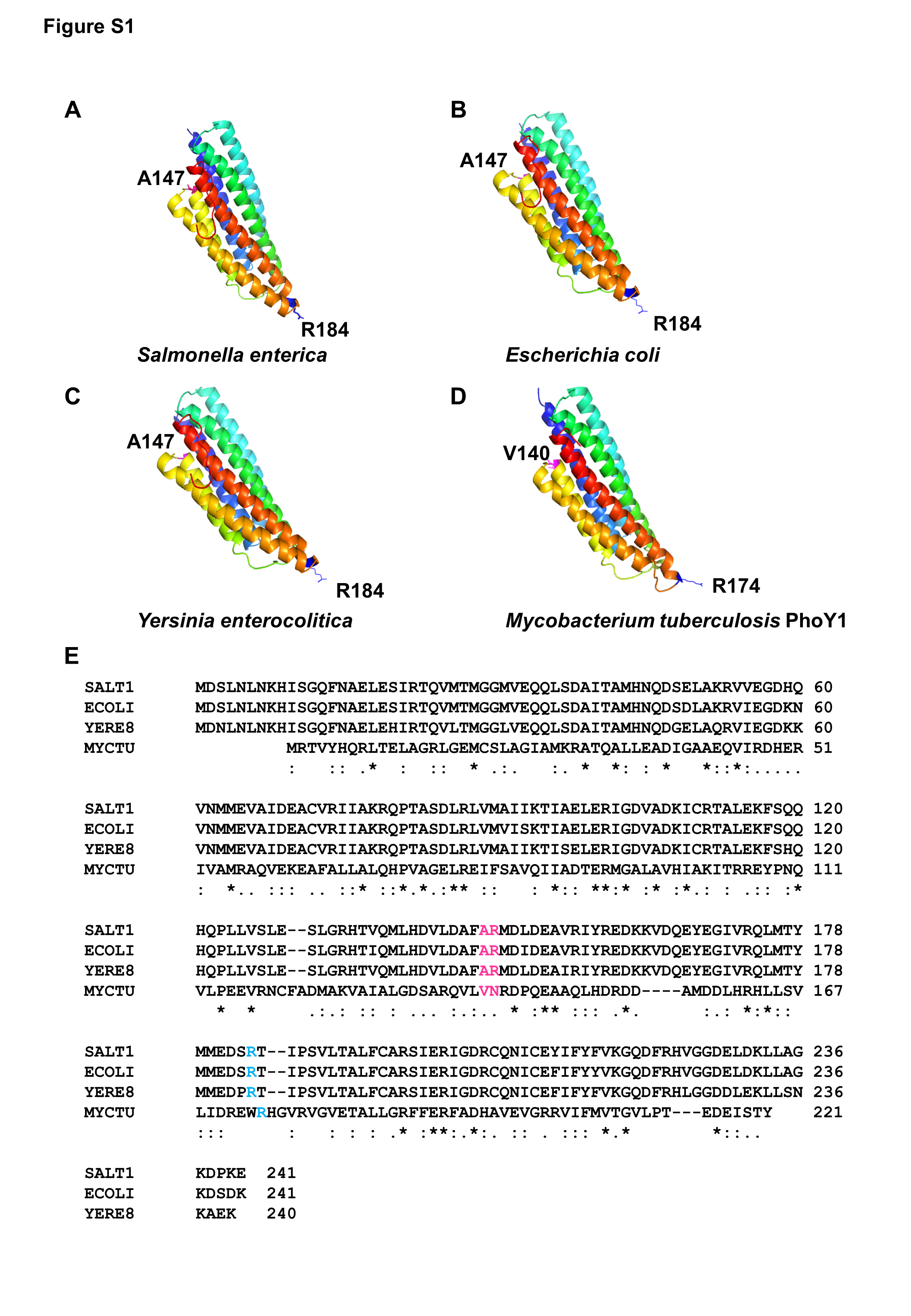

Supplement: FIG S1 [file mbio.00811-22-s0002.tif]

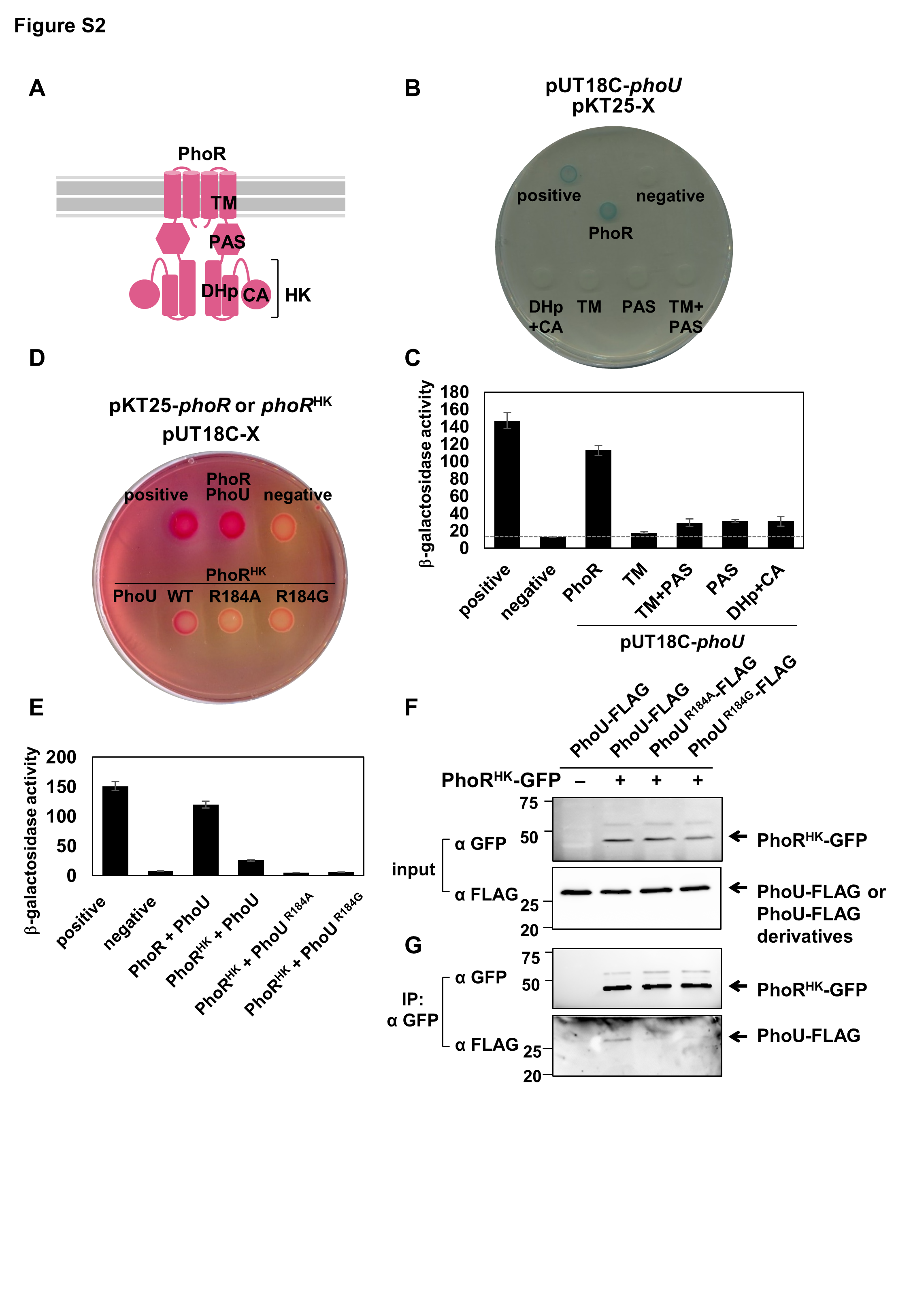

Supplement: FIG S2 [file mbio.00811-22-s0003.tif]

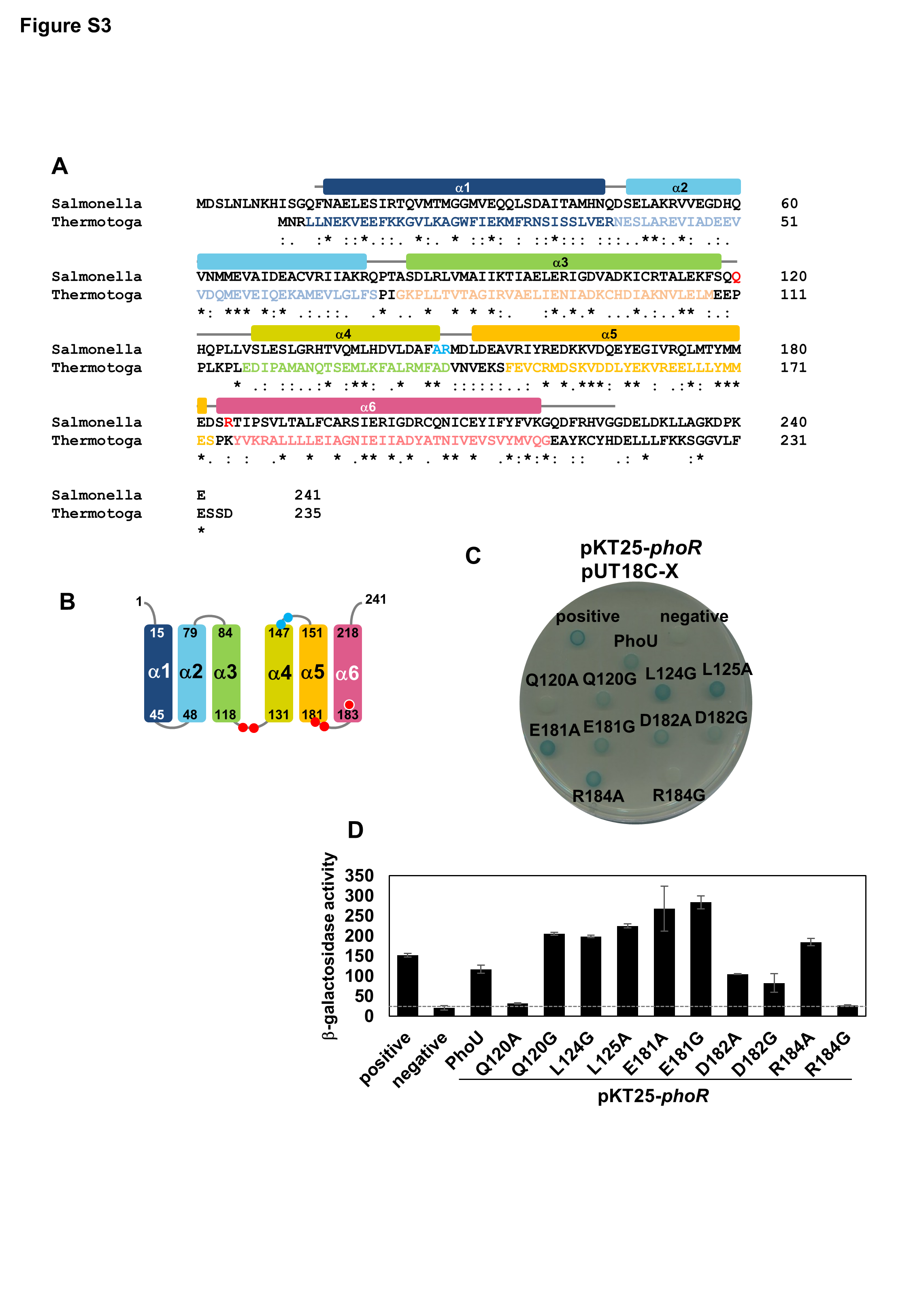

Supplement: FIG S3 [file mbio.00811-22-s0004.tif]

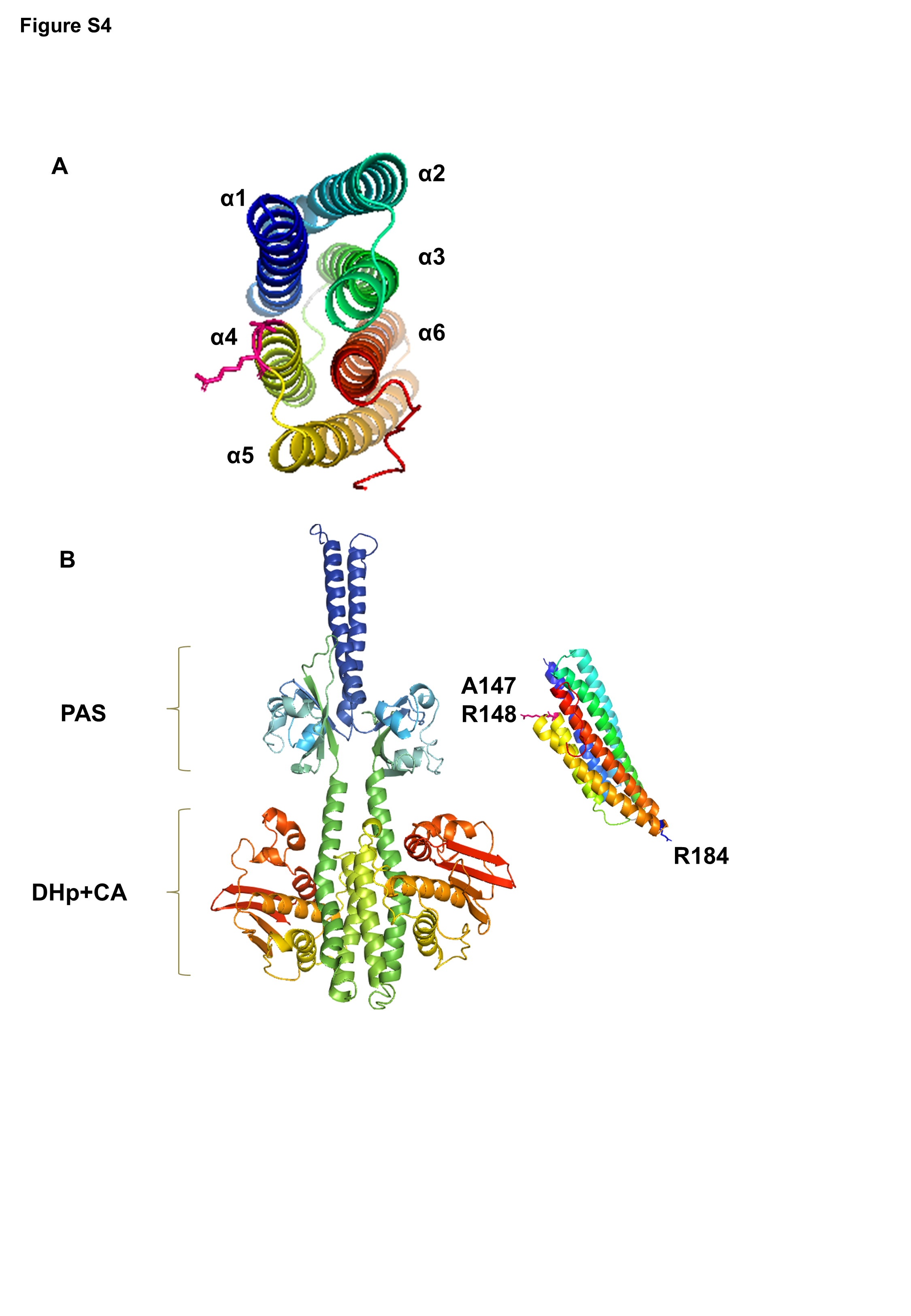

Supplement: FIG S4 [file mbio.00811-22-s0005.tif]

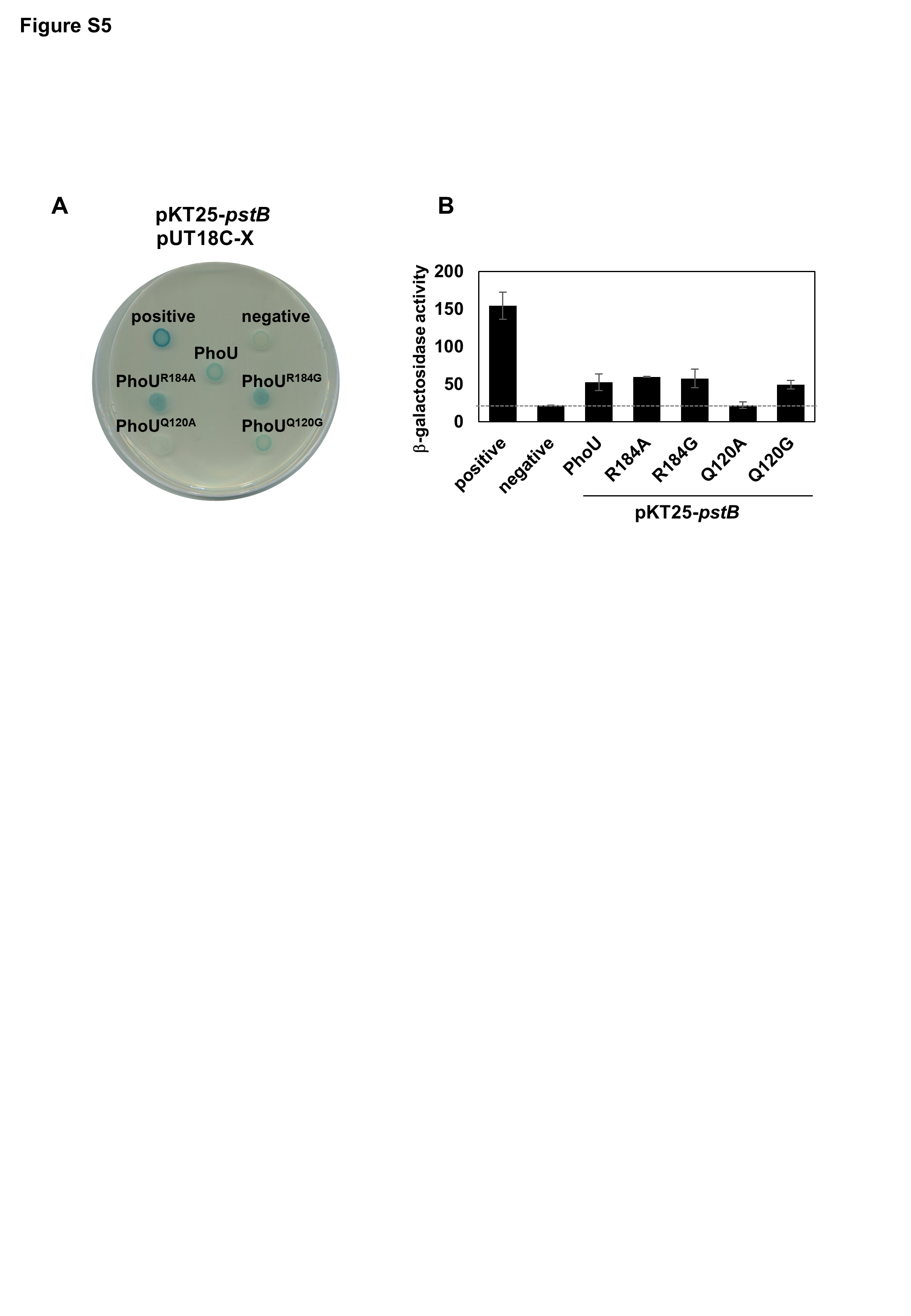

Supplement: FIG S5 [file mbio.00811-22-s0006.tif]

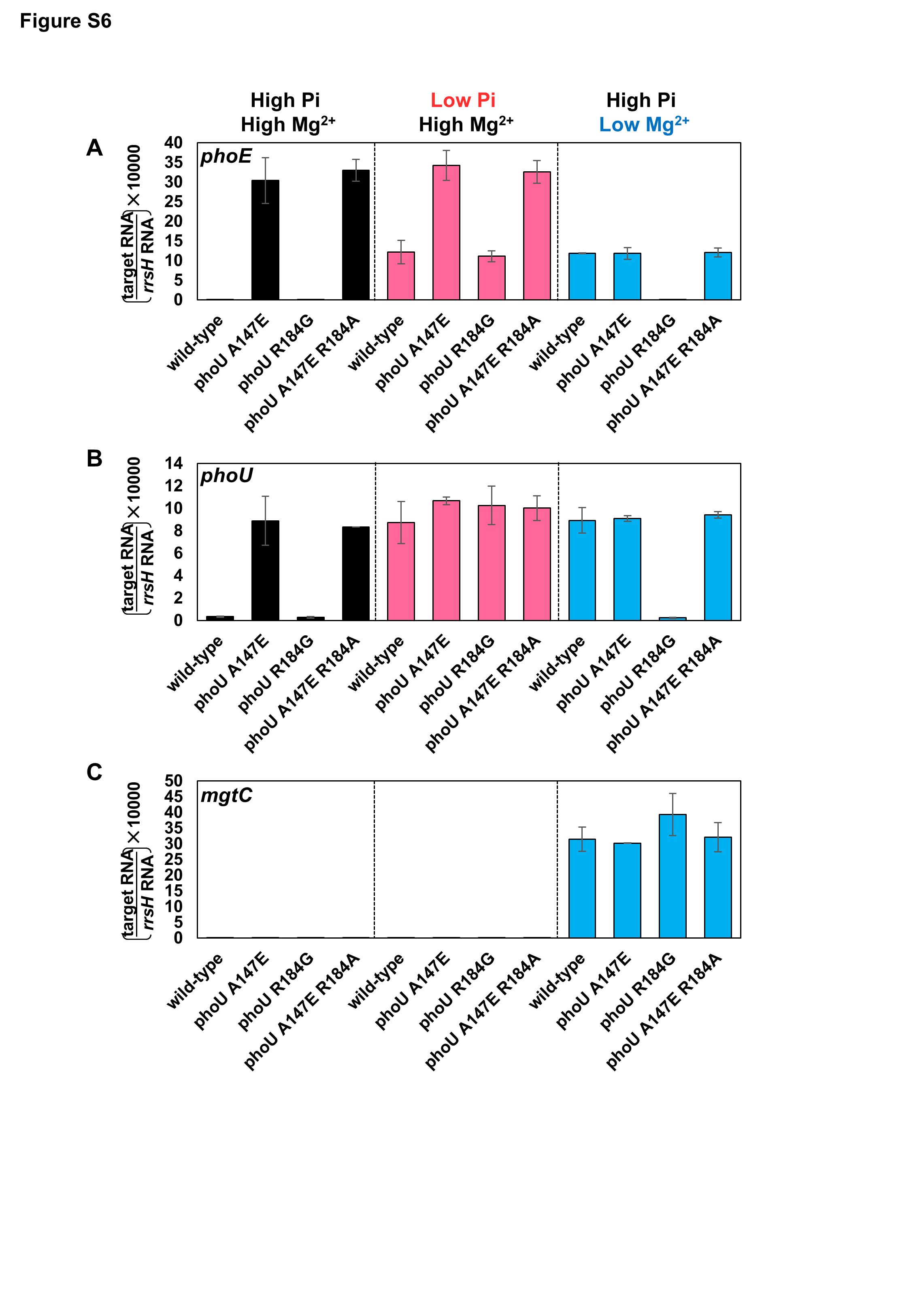

Supplement: FIG S6 [file mbio.00811-22-s0007.tif]

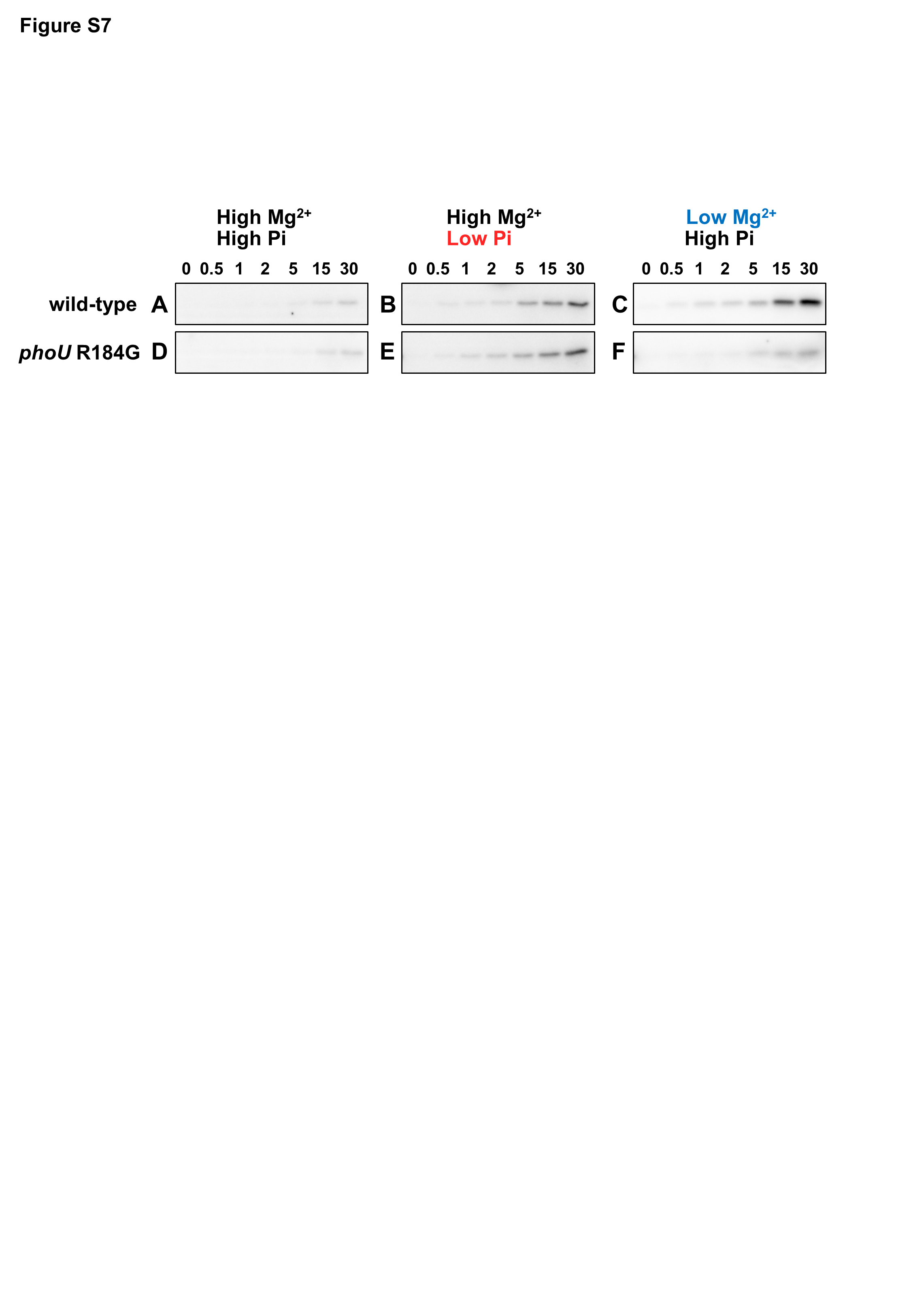

Supplement: FIG S7 [file mbio.00811-22-s0008.tif]
