# Supplementary material for: A Dual Regulatory Role of the PhoU Protein in Salmonella Typhimurium
Source: mBio. 2022 May 31;13(3):e00811-22. doi: 10.1128/mbio.00811-22 (PMC9239213; doi:10.1128/mbio.00811-22)
Supplement: TABLE S1 [file mbio.00811-22-s0009.docx]

**Table S1. Bacterial strains, plasmids, and oligonucleotides used in this study.**

1. **Bacterial strains and plasmids**

| **Name** | **Description** | **Reference** |
| --- | --- | --- |
| ***S. enteric* serovar Typhimurium** | | |
| 14028s | wild-type | (1) |
| MS7953s | *phoP7953*::Tn10 | (1) |
| SM097 | *phoU*::Cm^R^ | This study |
| SM101 | *phoU* | This study |
| EN1107 | *phoU*::Tet^R^ | This study |
| SM192 | *phoU*::Tet^R^ /pKD46 | This study |
| SM181 | *phoU* ^Gln 120 Ala^ | This study |
| SM179 | *phoU* ^Gln 120 Gly^ | This study |
| SM233 | *phoU* ^Arg 184 Ala^ | This study |
| SM235 | *phoU* ^Arg 184 Gly^ | This study |
| SM323 | *phoU* ^Ala 147 Glu^ | This study |
| SM427 | *phoU* ^Ala 147 Glu, Arg 184 Gly^ | This study |
| SM437 | *phoR*-8×Myc::Km^R^ | This study |
| SM439 | *phoU* ^Arg 184 Ala^, *phoR*-8×Myc::Km^R^ | This study |
| SM441 | *phoU* ^Arg 184 Gly^, *phoR*-8×Myc::Km^R^ | This study |
| SM454 | *phoR*-8×Myc | This study |
| SM455 | *phoU* ^Arg 184 Ala^, *phoR*-8×Myc | This study |
| SM456 | *phoU* ^Arg 184 Gly^, *phoR*-8×Myc | This study |
| SM401 | *phoU* Δ(480-1180)::Tet^R^ | This study |
| SM458 | *phoU*-FLAG | This study |
| SM459 | *phoU* ^Arg 184 Ala^-FLAG | This study |
| SM460 | *phoU* ^Arg 184 Gly^-FLAG | This study |
| EG19732 | 14028s/pUHE21 | (2) |
| EG19733 | 14028s/pUHE21*-mgtC* | (2) |
| GH10 | *phoU* ^Arg 184 Ala^/pUHE21 | This study |
| GH11 | *phoU* ^Arg 184 Gly^/pUHE21 | This study |
| GH14 | *phoU* ^Arg 184 Ala^/pUHE21-*mgtC* | This study |
| GH15 | *phoU* ^Arg 184 Gly^/pUHE21-*mgtC* | This study |
| SM461 | *phoR*-8×Myc/pBAD33 | This study |
| SM433 | *phoR*-8×Myc/pBAD33-*phoU*-C-His | This study |
| SM434 | *phoR*-8×Myc/pBAD33-*phoU* ^Arg 184 Ala^-C-His | This study |
| SM435 | *phoR*-8×Myc/pBAD33-*phoU* ^Arg 184 Gly^-C-His | This study |
| SM449 | *phoU*-FLAG/pTGFP-*phoR*^HK^ | This study |
| SM450 | *phoU* ^Arg 184 Ala^-FLAG/pTGFP-*phoR*^HK^ | This study |
| SM451 | *phoU* ^Arg 184 Gly^-FLAG/pTGFP-*phoR*^HK^ | This study |
| EL4 | *mgtC* | (2) |
| KK10 | *phoB* | (3) |
| ***Escherichia coli*** | | |
| DH5α | *fhuA2 lac(del)U169 phoA glnV44 Φ80' lacZ(del)M15 gyrA96 recA1 relA1 endA1 thi-1 hsdR17*. | (4) |
| SM087 | DH5α/pUT18-*phoU* | This study |
| SM309 | DH5α/pUT18c-*phoU* | This study |
| SM145 | DH5α/pUT18c-*phoU 1-44* | This study |
| SM139 | DH5α/pUT18c-*phoU 1-77* | This study |
| SM140 | DH5α/pUT18c-*phoU 1-119* | This study |
| SM146 | DH5α/pUT18c-*phoU 1-147* | This study |
| SM147 | DH5α/pUT18c-*phoU 1-181* | This study |
| SM185 | DH5α/pUT18c-*phoU 1-218* | This study |
| SM148 | DH5α/pUT18c-*phoU 1-120* | This study |
| SM125 | DH5α/pUT18c-*phoU* ^Gln 120 Ala^ | This study |
| SM126 | DH5α/pUT18c-*phoU* ^Gln 120 Gly^ | This study |
| SM105 | DH5α/pUT18c-*phoU* ^Leu 124 Gly^ | This study |
| SM112 | DH5α/pUT18c-*phoU* ^Leu 125 Ala^ | This study |
| SM114 | DH5α/pUT18c-*phoU* ^Glu 181 Ala^ | This study |
| SM115 | DH5α/pUT18c-*phoU* ^Glu 181 Gly^ | This study |
| SM116 | DH5α/pUT18c-*phoU* ^Asp 182 Ala^ | This study |
| SM117 | DH5α/pUT18c-*phoU* ^Asp 182 Gly^ | This study |
| SM118 | DH5α/pUT18c-*phoU* ^Arg 184 Ala^ | This study |
| SM217 | DH5α/pUT18c-*phoU* ^Arg 184 Gly^ | This study |
| SM312 | DH5α/pUT18c-*phoU* ^Ala 147 Glu^ | This study |
| SM313 | DH5α/pUT18c-*phoU* ^Ala 147 Lys^ | This study |
| SM310 | DH5α/pUT18c-*phoU* ^Arg 148 Ala^ | This study |
| SM107 | DH5α/pBAD33-*phoU*-C-His | This study |
| SM295 | DH5α/pBAD33-*phoU* ^Arg 184 Ala^-C-His | This study |
| SM297 | DH5α/pBAD33-*phoU* ^Arg 184 Gly^-C-His | This study |
| BTH101 | *F^-^, cya-854, recA1, endA1, gyrA96 (Nal^r^), thi1, hsdR17, spoT1, rfbD1, glnV44(AS)* | (5) |
| LJ18 | BTH101/pUT18-*mgtC*, pKT25-*mgtR* | (3) |
| LJ27 | BTH101/pUT18-*mgtC*, pKT25 | (3) |
| SM089 | BTH101/pUT18-*phoU*, pKT25-*phoR* | This study |
| GH34 | BTH101/pUT18-*phoU*, pKT25-*phoR_TM_(1-60)* | This study |
| GH35 | BTH101/pUT18-*phoU*, pKT25-*phoR_PAS_(61-180)* | This study |
| GH33 | BTH101/pUT18-*phoU*, pKT25-*phoR DHp+CA (181-431)* | This study |
| GH36 | BTH101/pUT18-*phoU*, pKT25-*phoR_TM+PAS_(1-180)* | This study |
| SM169 | BTH101/pUT18c-*phoU* 1-44, pKT25-*phoR* | This study |
| SM170 | BTH101/pUT18c-*phoU* 1-77, pKT25-*phoR* | This study |
| SM171 | BTH101/pUT18c-*phoU* 1-119, pKT25-*phoR* | This study |
| SM172 | BTH101/pUT18c-*phoU* 1-147, pKT25-*phoR* | This study |
| SM173 | BTH101/pUT18c-*phoU* 1-181, pKT25-*phoR* | This study |
| SM186 | BTH101/pUT18c-*phoU* 1-218, pKT25-*phoR* | This study |
| SM174 | BTH101/pUT18c-*phoU* 1-120, pKT25-*phoR* | This study |
| SM127 | BTH101/pUT18c-*phoU* ^Gln 120 Ala^, pKT25-*phoR* | This study |
| SM128 | BTH101/pUT18c-*phoU* ^Gln 120 Gly^, pKT25-*phoR* | This study |
| SM104 | BTH101/pUT18c-*phoU* ^Leu 124 Gly^, pKT25-*phoR* | This study |
| SM113 | BTH101/pUT18c-*phoU* ^Leu 125 Ala^, pKT25-*phoR* | This study |
| SM119 | BTH101/pUT18c-*phoU* ^Glu 181 Ala^, pKT25-*phoR* | This study |
| SM120 | BTH101/pUT18c-*phoU* ^Glu 181 Gly^, pKT25-*phoR* | This study |
| SM121 | BTH101/pUT18c-*phoU* ^Asp 182 Ala^, pKT25-*phoR* | This study |
| SM122 | BTH101/pUT18c-*phoU* ^Asp 182 Gly^, pKT25-*phoR* | This study |
| SM123 | BTH101/pUT18c-*phoU* ^Arg 184 Ala^, pKT25-*phoR* | This study |
| SM219 | BTH101/pUT18c-*phoU* ^Arg 184 Gly^, pKT25-*phoR* | This study |
| SM315 | BTH101/pUT18c-*phoU* ^Ala 147 Glu^, pKT25-*phoR* | This study |
| SM314 | BTH101/pUT18c-*phoU* ^Ala 147 Lys^, pKT25-*phoR* | This study |
| SM311 | BTH101/pUT18c-*phoU* ^Arg 148 Ala^, pKT25-*phoR* | This study |
| SM252 | BTH101/pUT18-*phoU*, pKT25-*pstB* | This study |
| SM253 | BTH101/pUT18c-*phoU* ^Gln 120 Ala^, pKT25-*pstB* | This study |
| SM254 | BTH101/pUT18c-*phoU* ^Gln 120 Gly^, pKT25-*pstB* | This study |
| SM293 | BTH101/pUT18c-*phoU* ^Arg 184 Ala^, pKT25-*pstB* | This study |
| SM294 | BTH101/pUT18c-*phoU* ^Arg 184 Gly^, pKT25-*pstB* | This study |
| **Plasmids** | | |
| pUHE21-2lacI^q^ | rep_pMBI_ Ap^R^ *lacI*^q^ | (6) |
| pBAD33 | pACYC184 *ori* Cm^R^ | (7) |
| pKD3 | repR_6Kγ_ Ap^R^ FRT Cm^R^ FRT | (8) |
| pKD46 | rep_pSC101_^ts^ Ap^R^ P*_araBAD_* γ β exo | (8) |
| pCP20 | rep_pSC101_^ts^ Ap^R^ Cm^R^ *cI857* λP_R_*flp* | (8) |
| pBOP508 | repR_R6K_ Ap^R^ 8×myc FRT Km^R^ FRT | (9) |
| pUT18 | p_lac_ ColEI *ori* Ap^R^ | (10) |
| pUT18c | p_lac_ ColEI *ori* Ap^R^ | (10) |
| pKT25 | p_lac_ p15A*ori* Km^R^ | (10) |
| pTGFP | ColE1 *ori* Ap^R^ 'gfp | (11, 12) |

**(B) Primers used in this study.**

| **Name** | **Sequence (from 5’ to 3’)*** |  |
| --- | --- | --- |
| **Knockout, deletion, tagging** | |  |
| KHU763 | ACT ATA TCA CCG GTC GTT ACG GTT AAT TCA GGA GTG CGT ATG TAG GCT GGA GCT GCT TCG | *phoU* deletion Cm^R^ cassette insertion |
| KHU764 | GGC CCG GTA AGC GCA GCG CCA CCG GGC AAA AGA ATG AGA TCA TAT GAA TAT CCT CCT TAG | *phoU* deletion Cm^R^ cassette insertion |
| KHU1012 | TCA ACG CCG AAC TGG AAA GCA TCC GCA CTC AGG TAA TGA CTT AAG ACC CAC TTT CAC ATT TAA G | *phoU* Tet^R^ cassette insertion |
| KHU1013 | GCA GCT TAT CCA GCT CAT CGC CGC CGA CGT GAC GGA AAT CCT AAG CAC TTG TCT CCT GTT TAC | *phoU* Tet^R^ cassette insertion |
| KU452 | TGA GCT GGA TAA GCT GCT GGC GGG GAA AGA TCC GAA AGA GAT CGG ATC CAG AAT TCG TGA T | *phoR*-8×Myc Km^R^ insertion |
| KU453 | GGC CCG GTA AGC GCA GCG CCA CCG GGC AAA AGA ATG AGA TGA GCT CGA TCC GTC GAC C | *phoR*-8×Myc Km^R^ insertion |
| KU458 | ACC GTT GCC AGA ATA TCT GCG AAT ACA TCT TCT ACT TCG TTT AAG ACC CAC TTT CAC ATT TAA G | *phoU* Δ(480-1180) Tet^R^ cassette insertion |
| KU459 | AAC TGG AAG CGA TCG CGG GCG CAA ATA CCC TGC TGG AGA GCT AAG CAC TTG TCT CCT GTT TAC | *phoU* Δ(480-1180) Tet^R^ cassette insertion |
| KU464 | GGG GAA AGA TCC GAA AGA GGA CTA CAA GGA CGA CGA TGA CAA GTA AAT CTC ATT CTT TTG | *phoU-*FLAG template PCR |
| KU465 | GGG GAA AGA TCC GAA AGA GGA CTA CAA GGA CGA CGA TGA CAA GTA AAT CTC ATT CTT TTG | *phoU-*FLAG template PCR |
| KU462 | GAC AAG AAA GTT GAC CAG GA | *phoU-*FLAG template PCR |
| KU463 | TTA AAG GCG TAA AGC TCT AC | *phoU-*FLAG template PCR |
| **Cloning** | |  |
| KHU153 | CGG GAT CCG CTG GAA CGG CTG TCA TGG AAA | *phoR*-BamHI |
| KHU154 | GGG GTA CCC CAT CGC TAT TTT TGG CAA TTA | *phoR*-KpnI |
| KHU155 | CGG GAT CCG GAC AGT CTG AAC CTT AAT AAA | *phoU*-BamHI |
| KHU156 | GGG GTA CCC CCT CTT TCG GAT CTT TCC CCG | *phoU*-KpnI |
| KHU767 | CCC AAG CTT TTA GTG GTG ATG GTG ATG ATG CTC TTT CGG ATC TTT CCC CG | *phoU*-His-HindIII |
| KHU768 | GCT CTA GAT AAT TCA GGA GTG CGT AAT GGA CAG TCT GAA CCT TAA | *phoU*-XbaI |
| KHU823 | GGG GTA CCC CGT GCA TGG CGG TGA TAG CGT | *phoU* helix1 |
| KHU824 | GGG GTA CCC CGG CGA TAA TAC GCA CGC AGG | *phoU* helix2 |
| KHU825 | GGG GTA CCC CCT GGG AGA ATT TCT CCA GCG | *phoU* helix3 |
| KHU826 | GGG GTA CCC CCG CGA ACG CAT CCA GCA CGT | *phoU* helix4 |
| KHU827 | GGG GTA CCC CTT CCA TCA TGT AGG TCA TCA | *phoU* helix5 |
| KHU828 | GGG GTA CCC CCT TCA CGA AGT AGA AGA TGT | *phoU* helix6 |
| KHU981 | GGG GTA CCC CCT GCT GGG AGA ATT TCT CCA | *phoU*120-KpnI |
| **Site-directed mutagenesis** | |  |
| KHU1014 | GAC TAT ATC ACC GGT CGT TAC | *phoU* amino acid mutation PCR forward |
| KHU1015 | GCA TCT GTA CCT GTA GGC CCG | *phoU* amino acid mutation PCR reverse |
| KU68 | GTG CTG GAT GCG TTC GAG CGC ATG GAT CTC GAC | *phoU* ^A147E^ substitution |
| KU69 | GTC GAG ATC CAT GCG CTC GAA CGC ATC CAG CAC | *phoU* ^A147E^ substitution |
| KU70 | GTG CTG GAT GCG TTC AAG CGC ATG GAT CTC GAC | *phoU* ^A147K^ substitution |
| KU71 | GTC GAG ATC CAT GCG CTT GAA CGC ATC CAG CAC | *phoU* ^A147K^ substitution |
| KU72 | CTG GAT GCG TTC GCG GCC ATG GAT CTC GAC GAA | *phoU* ^R148A^ substitution |
| KU73 | TTC GTC GAG ATC CAT GGC CGC GAA CGC ATC CAG | *phoU* ^R148A^ substitution |
| KHU777 | GGA AGA CTC GGC CAC CAT TCC CA | *phoU* ^R184A^ substitution |
| KHU778 | TGG GAA TGG TGG CCG AGT CTT CC | *phoU* ^R184A^ substitution |
| KHU779 | GGA AGA CTC GGG CAC CAT TCC CA | *phoU* ^R184G^ substitution |
| KHU780 | TGG GAA TGG TGC CCG AGT CTT CC | *phoU* ^R184G^ substitution |
| KHU793 | ATT CTC CCA GGC GCA TCA GCC GC | *phoU* ^Q120A^ substitution |
| KHU794 | GCG GCT GAT GCG CCT GGG AGA AT | *phoU* ^Q120A^ substitution |
| KHU795 | ATT CTC CCA GGG GCA TCA GCC GC | *phoU* ^Q120G^ substitution |
| KHU796 | GCG GCT GAT GCC CCT GGG AGA AT | *phoU* ^Q120G^ substitution |
| KHU769 | CTA CAT GAT GGC AGA CTC GCG CA | *phoU* ^E181A^ substitution |
| KHU770 | TGC GCG AGT CTG CCA TCA TGT AG | *phoU* ^E181A^ substitution |
| KHU771 | CTA CAT GAT GGG AGA CTC GCG CA | *phoU* ^E181G^ substitution |
| KHU772 | TGC GCG AGT CTC CCA TCA TGT AG | *phoU* ^E181G^ substitution |
| KHU773 | CAT GAT GGA AGC CTC GCG CAC CA | *phoU* ^D182A^ substitution |
| KHU774 | TGG TGC GCG AGG CTT CCA TCA TG | *phoU* ^D182A^ substitution |
| KHU775 | CAT GAT GGA AGG CTC GCG CAC CA | *phoU* ^D182G^ substitution |
| KHU776 | TGG TGC GCG AGC CTT CCA TCA TG | *phoU* ^D182G^ substitution |
| KHU783 | TCA GCC GCT GGC GGT GAG TCT GG | *phoU* ^L125A^ substitution |
| KHU784 | CCA GAC TCA CCG CCA GCG GCT GA | *phoU* ^L125A^ substitution |
| KHU787 | GCA TCA GCC GGG GCT GGT GAG TC | *phoU* ^L124G^ substitution |
| KHU788 | GAC TCA CCA GCC CCG GCT GAT GC | *phoU* ^L124G^ substitution |
| **For qRT-PCR** | |  |
| KHQ015 | CGCTGGGAAGCTGAGTTTG | *phoE* qPCR-F |
| KHQ016 | CCAGGCCTCAACATCGTACA | *phoE* qPCR-R |
| KHQ097 | CATATTTCCGGCCAGTTCAAC | *phoU* qPCR-F |
| KHQ098 | CGGTGATAGCGTCAGAAAG | *phoU* qPCR-R |
| 6970 | CCAGCAGCCGCGGTAAT | *rrsH* qPCR -F |
| 6971 | TTTACGCCCAGTAATTCCGATT | *rrsH* qPCR -R |
| 7530 | CAGCCCGCGCACATTC | *mgtC* qPCR -F |
| 7531 | CAGCCCGCGCACATTC | *mgtC* qPCR -R |

**References**

1. Fields PI, Swanson RV, Haidaris CG, Heffron F. 1986. Mutants of *Salmonella* *typhimurium* that cannot survive within the macrophage are avirulent. Proc Natl Acad Sci U S A 83:5189-93.

2. Lee EJ, Pontes MH, Groisman EA. 2013. A Bacterial Virulence Protein Promotes Pathogenicity by Inhibiting the Bacterium's Own F1Fo ATP Synthase. Cell 154:146-56.

3. Choi S, Choi E, Cho YJ, Nam D, Lee J, Lee EJ. 2019. The Salmonella virulence protein MgtC promotes phosphate uptake inside macrophages. Nat Commun 10:3326.

4. Taylor RG, Walker DC, McInnes RR. 1993. *E. coli* host strains significantly affect the quality of small scale plasmid DNA preparations used for sequencing. Nucleic Acids Res 21:1677-8.

5. Karimova G, Pidoux J, Ullmann A, Ladant D. 1998. A bacterial two-hybrid system based on a reconstituted signal transduction pathway. Proc Natl Acad Sci U S A 95:5752-6.

6. Soncini FC, Vescovi EG, Groisman EA. 1995. Transcriptional autoregulation of the *Salmonella typhimurium* *phoPQ* operon. J Bacteriol 177:4364-71.

7. Guzman LM, Belin D, Carson MJ, Beckwith J. 1995. Tight regulation, modulation, and high-level expression by vectors containing the arabinose PBAD promoter. J Bacteriol 177:4121-30.

8. Datsenko KA, Wanner BL. 2000. One-step inactivation of chromosomal genes in *Escherichia coli* K-12 using PCR products. Proc Natl Acad Sci U S A 97:6640-5.

9. Cho BK, Knight EM, Palsson BO. 2006. PCR-based tandem epitope tagging system for Escherichia coli genome engineering. Biotechniques 40:67-72.

10. Karimova G, Ullmann A, Ladant D. 2001. Protein-protein interaction between *Bacillus stearothermophilus* tyrosyl-tRNA synthetase subdomains revealed by a bacterial two-hybrid system. J Mol Microbiol Biotechnol 3:73-82.

11. Lee EJ, Groisman EA. 2012. Tandem Attenuators Control Expression of the *Salmonella* *mgtCBR* Virulence Operon. Mol Microbiol 86:212-24.

12. Choi E, Han Y, Park S, Koo H, Lee JS, Lee EJ. 2021. A Translation-Aborting Small Open Reading Frame in the Intergenic Region Promotes Translation of a Mg(2+) Transporter in Salmonella Typhimurium. mBio 12.
