# Supplementary material for: A Dual Regulatory Role of the PhoU Protein in Salmonella Typhimurium
Source: mBio. 2022 May 31;13(3):e00811-22. doi: 10.1128/mbio.00811-22 (PMC9239213; doi:10.1128/mbio.00811-22)
Supplement: TEXT S1 [file mbio.00811-22-s0001.docx]

**Supplemental Information**

**A dual regulatory role of the PhoU protein in *Salmonella* Typhimurium**

**Running Title:** Dual regulatory role of the PhoU protein in *Salmonella*

Soomin Choi^a,b^, Gyunghwa Jeong^a,b^, Eunna Choi^a^, Eun-Jin Lee^a,^#

^a^ Department of Life Sciences, School of Life Sciences and Biotechnology, Korea University, Seoul, South Korea

^b^ These authors contributed equally to this work

Correspondence
Eun-Jin Lee, eunjinlee@korea.ac.kr

**This file includes:**

Supplemental text (Text S1)

Supplemental references

# Text S1. Supplemental materials and methods

**Plasmid construction**

For the bacterial two-hybrid assays, the plasmids pUT18-*phoU*, pUT18c-*phoU*, pUT18c-*phoU* 1-44, pUT18c-*phoU* 1-77, pUT18c-*phoU* 1-119, pUT18c-*phoU* 1-147, pUT18c-*phoU* 1-181, pUT18c-*phoU* 1-218, pUT18c-*phoU* 1-120, pUT18c-*phoU* ^Gln 120 Ala^, pUT18c-*phoU* ^Gln 120 Gly^, pUT18c-*phoU* ^Leu 124 Gly^, pUT18c-*phoU* ^Leu 125 Ala^, pUT18c-*phoU* ^Glu 181 Ala^, pUT18c-*phoU* ^Glu 181 Gly^, pUT18c-*phoU* ^Asp 182 Ala^, pUT18c-*phoU* ^Asp 182 Gly^ , pUT18c-*phoU* ^Arg 184 Ala^, pUT18c-*phoU* ^Arg 184 Gly^, pUT18c-*phoU* ^Ala 147 Glu^, pUT18c-*phoU* ^Ala 147 Lys^, and pUT18c-*phoU* ^Arg 148 Ala^ were constructed as follows: DNA fragments corresponding to the *phoU*, *phoU* 1-44, *phoU* 1-77, *phoU* 1-119, *phoU* 1-147, *phoU* 1-181, *phoU* 1-218, *phoU* 1-120, *phoU* ^Leu 124 Gly^*, phoU* ^Leu 125 Ala^, *phoU* ^Glu 181 Ala^, *phoU* ^Glu 181 Gly^, *phoU* ^Asp 182 Ala^, *phoU* ^Asp 182 Gly^ , *phoU* ^Ala 147 Glu^, *phoU* ^Ala 147 Lys^, and *phoU* ^Arg 148 Ala^ were ampliﬁed by PCR using the primer pairs, KHU155/KHU156 (for *phoU*), KHU155/KHU823 (for *phoU* 1-44), KHU155/KHU824 (for *phoU* 1-77), KHU155/KHU825 (for *phoU* 1-119), KHU155/KHU826 (for *phoU* 1-147), KHU155/KHU827 (for *phoU* 1-181), KHU155/KHU828 (for *phoU* 1-218), KHU155/KHU981 (for *phoU* 1-120), KHU155/KHU788 and KHU787/KHU156 (for *phoU* ^Leu 124 Gly^), KHU155/KHU784 and KHU783/KHU156 (for *phoU* ^Leu 125 Ala^), KHU155/KHU770 and KHU769/KHU156 (for *phoU* ^Glu 181 Ala^), KHU155/KHU772 and KHU771/KHU156 (for *phoU* ^Glu 181 Gly^), KHU155/KHU774 and KHU773/KHU156 (for *phoU* ^Asp 182 Ala^), KHU155/KHU776 and KHU775/KHU156 (for *phoU* ^Asp 182 Gly^), KHU155/KU69 and KU68/KHU156 (for *phoU* ^Ala 147 Glu^), KHU155/KU71 and KU70/KHU156 (for *phoU* ^Ala 147 Lys^), and KHU155/KU73 and KU72/KHU156 (for *phoU* ^Arg 148 Ala^), using 14028s genomic DNA as a template. For pUT18c-*phoU* ^Gln 120 Ala^, pUT18c-*phoU* ^Gln 120 Gly^, pUT18c-*phoU* ^Arg 184 Ala^, and pUT18c-*phoU* ^Arg 184 Gly^, DNA fragments were ampliﬁed by PCR using the primer pairs KHU155/KHU156 and SM181, SM179, SM233, and SM235 genomic DNA as templates. After puriﬁcation, the PCR products were digested with BamHI and KpnI and cloned into pKT25, pUT18 or pUT18c plasmids digested with the same enzymes.

The plasmids pBAD33-*phoU*-C-His, pBAD33-*phoU* ^Arg 184 Ala^-C-His, and pBAD33-*phoU* ^Arg 184 Gly^-C-His were constructed as follows: PCR fragments corresponding to the *phoU, phoU* ^Arg 184 Ala^ and *phoU* ^Arg 184 Gly^ genes were generated by PCR with the primer pairs KHU767/KHU768 using 14028s (for *phoU*), SM233 (for *phoU* ^Arg 184 Ala^), and SM235 (for *phoU* ^Arg 184 Gly^) genomic DNAs as templates respectively. The amplified DNA fragments were digested with XbaI and HindIII (for *phoU*-C-His, *phoU* ^Arg 184 Ala^-C-His, and *phoU* ^Arg 184 Gly^-C-His), and cloned into pBAD33 digested with the same enzymes.

The pTGFP-*phoR*^HK^ plasmid was constructed as follows: a PCR fragment corresponding to PhoR HK domain (181-431 aa) was generated by PCR with primers KU451 and KHU451 using 14028s genomic DNA as a template. The amplified DNA fragments were digested with EcoRI and BamHI and cloned into pTGFP digested with the same enzymes. The sequences of the resulting constructs were veriﬁed by DNA sequencing.

**Construction of strains with chromosomal substitutions in the *phoU* gene**

To generate strains with chromosomal mutations in the *phoU* coding region, we used the fusaric acid-based counter selection method as described previously (1). First, we introduced Tet^R^ cassettes in the coding region of the *phoU* gene as follows: we generated a PCR product harboring the *tetRA* gene using primers KHU1012/KHU1013 and MS7953s chromosomal DNA as a template. The PCR product was purified using a QIAquick PCR purification kit (QIAGEN) and used to electroporate the 14028s chromosome containing plasmid pKD46 (2). The resulting *phoU*::Tet^R^ (ENC1107) strain containing plasmid pKD46 were kept at 30°C. Then, we replaced the *tetRA* cassettes by preparing DNA fragments carrying various nucleotide substitutions in the *phoU* gene by a two-step PCR process. For the first PCR reaction, we used two primer pairs KHU1014/KHU778 and KHU777/KHU1015 (for 184^th^ arginine codon to alanine), KHU1014/KHU780 and KHU779/KHU1015 (for 184^th^ arginine codon to glycine), KHU1014/KHU794 and KHU793/KHU1015 (for 120^th^ glutamine codon to alanine), KHU1014/KHU796 and KHU795/KHU1015 (for 120^th^ glutamine codon to glycine), and KHU1014/KU69 and KU68/KHU1015 (for 147^th^ alanine codon to glutamate), and 14028s genomic DNA as a template. For the second PCR reaction, we mixed the two PCR products from the first PCR reaction as templates and amplified DNA fragments using primers KHU1014 and KHU1015. The resulting PCR products were purified and integrated into the EN1107 (*phoU*::Tet^R^) chromosome harboring pKD46 and selected against Tet^R^ with media containing fusaric acid to generate SM233 (*phoU* ^Arg 184 Ala^), SM235 (*phoU* ^Arg 184 Gly^), SM181 (*phoU* ^Gln 120 Ala^), SM179 (*phoU* ^Gln 120 Gly^), and SM323 (*phoU* ^Ala 147 Glu^), tetracycline-sensitive, ampicillin-sensitive (Tet^S^ Amp^S^) chromosomal mutants, respectively. To generate the *phoU* ^Ala 147 Glu, Arg 184 Gly^ strain, we used two primer pairs KHU1014/KU69 and KU68/KHU1015 (for 147^th^ alanine codon to glutamate) and SM235 (*phoU* ^Arg 184 Gly^) genomic DNA as a template. Then, we mixed the two PCR products from the previous PCR reaction as templates and amplified DNA fragments using primers KHU1014 and KHU1015. The resulting PCR products were purified and integrated into the EN1107 (*phoU*::Tet^R^) chromosome harboring pKD46 and selected against Tet^R^ with media containing fusaric acid to generate SM427 (*phoU* ^Ala 147 Glu, Arg 184 Gly^), a tetracycline-sensitive, ampicillin-sensitive (Tet^S^ Amp^S^) chromosomal mutant.

To generate strains with the C-terminally FLAG-tagged *phoU* gene or its derivatives, we introduced a Tet^R^ cassette near the C-terminus of the *phoU* gene (from 480 to 1180 nt from the start codon of the *phoU* gene) as follows: we generated a PCR product harboring the *tetRA* gene using primers KU458/KU459 and MS7953s chromosomal DNA as a template. The PCR product was purified and used to electroporate the 14028s chromosome containing plasmid pKD46 (2). The resulting *phoU* Δ(480-1180)::Tet^R^ (SM401) strain containing plasmid pKD46 were kept at 30°C. Then, we replaced the Tet^R^ cassettes by preparing DNA fragments carrying various nucleotide substitutions with the C-terminal FLAG tag at the *phoU* gene by a two-step PCR process. For the first PCR reaction, we used two primer pairs KU462/KU465 and KU464/KU463 using 14028s (for *phoU*-FLAG), SM233 (for *phoU* ^Arg 184 Ala^-FLAG), and SM235 (for *phoU* ^Arg 184 Gly^-FLAG) genomic DNA as templates. For the second PCR reaction, we mixed the two PCR products from the first PCR reaction as templates and amplified DNA fragments using primers KU462 and KU463. The resulting PCR products were purified and integrated into the SM401 (*phoU*Δ(480-1180)::Tet^R^) chromosome harboring pKD46 and selected against Tet^R^ with media containing fusaric acid to generate SM458 (*phoU*-FLAG), SM459 (*phoU* ^Arg 184 Ala^-FLAG), and SM460 (*phoU* ^Arg 184 Gly^-FLAG), tetracycline-sensitive, ampicillin-sensitive (Tet^S^ Amp^S^) chromosomal mutants, respectively. The presence of the expected nucleotide substitutions was verified by DNA sequencing.

**Construction of a strain with the chromosomal *phoU* deletion**

A *Salmonella* strain deleted for the *phoU* gene was generated by the one-step gene inactivation method (2). A Cm^R^ cassette for the *phoU* gene was PCR amplified from plasmid pKD3 using primers KHU763/KHU764 and the resulting PCR product was integrated into the 14028s chromosome to generate SM097 (*phoU*::Cm^R^). The *phoU* strain (SM101) was generated by removing Cm^R^ cassette from SM097 via plasmid pCP20 as described (2).

**Construction of strains with the C-terminally Myc-tagged *phoR* gene at its chromosomal location**

*Salmonella* strains with C-terminal myc-tag fused to the *phoR* gene were generated by the PCR-based tandem epitope tagging system (3). A Km^R^ cassette for the *phoR*-8×myc genes was PCR amplified from plasmid pBOP508 using KU452 and KU453 primers. The resulting PCR product was integrated into the 14028s, SM233, or SM235 chromosomes to generate SM437 (*phoR*-8×myc::Km^R^), SM439 (*phoU* ^Arg 184 Ala^, *phoR*-8×myc::Km^R^), or SM441 (*phoU* ^Arg 184 Gly^, *phoR*-8×myc::Km^R^), respectively. The SM454 (*phoR*-8×myc), SM455 (*phoU* ^Arg 184 Ala^, *phoR*-8×myc), or SM456 (*phoU* ^Arg 184 Gly^, *phoR*-8×myc) strains were generated by removing Km^R^ cassettes from SM437, SM439, or SM441 using plasmid pCP20 as described (2).

**Supplemental references**

1. Lee EJ, Groisman EA. 2010. An antisense RNA that governs the expression kinetics of a multifunctional virulence gene. Mol Microbiol 76:1020-33.

2. Datsenko KA, Wanner BL. 2000. One-step inactivation of chromosomal genes in *Escherichia coli* K-12 using PCR products. Proc Natl Acad Sci U S A 97:6640-5.

3. Cho BK, Knight EM, Palsson BO. 2006. PCR-based tandem epitope tagging system for Escherichia coli genome engineering. Biotechniques 40:67-72.
